# Microbial Diversity and Genome Analyses Provide Insights into the Role of *Pseudomonas marginalis* A39 in Improving Drought Tolerance in *Pinus sylvestris* var. *mongolica*

**DOI:** 10.3390/microorganisms14071544

**Published:** 2026-07-15

**Authors:** Qian Song, Xiaoshuang Song, Xun Deng, Jian Liang

**Affiliations:** 1 State Key Laboratory of Plateau Ecology and Agriculture, Qinghai University, Xining 810016, China; 2023990003@qhu.edu.cn; 2 Institute of Forestry Protection, Heilongjiang Forestry Academy, Harbin 150040, China

**Keywords:** forest seedlings, functional annotation, oxidative stress, plant growth-promoting rhizobacteria, rhizosphere microbiome, soil biochemical properties

## Abstract

Drought severely constrains the growth of *Pinus sylvestris* var. *mongolica*, but the role of plant growth-promoting rhizobacteria in forest drought responses remains insufficiently understood. This study evaluated whether *Pseudomonas marginalis* A39 alleviates drought stress through plant physiological regulation, soil functional improvement, and rhizosphere bacterial community shifts. Strain A39 was first assessed for drought tolerance and plant growth-promoting traits in vitro. A greenhouse pot experiment was then conducted under graded drought levels with or without A39 inoculation, followed by analyses of plant growth, physiological traits, soil nutrients, enzyme activities, rhizosphere bacterial communities, whole-genome features, and drought-responsive gene expression. A39 tolerated low water potential and showed multiple growth-promoting traits. Under severe drought, A39 inoculation increased seedling height, ground diameter, aboveground dry weight, and underground dry weight by 83.58%, 47.69%, 37.14%, and 41.67%, respectively. It also increased total chlorophyll content and CAT activity by 30.38% and 40.20%, while reducing MDA and proline accumulation by 33.71% and 35.40%, respectively. A39 improved soil nutrient availability, with available nutrients increasing by more than 35%, and altered rhizosphere bacterial communities, with enrichment of taxa such as *Pseudarthrobacter* and *Pseudomonas*. Soil sucrase, available potassium, and total potassium were key factors associated with bacterial community variation. Genome annotation and qRT-PCR analysis identified candidate genes potentially related to nutrient acquisition, phytohormone production, oxidative stress defense, and osmotic adaptation. These results indicate that A39 inoculation is associated with improved drought performance of *P. sylvestris* var. *mongolica*, supporting its further evaluation as a candidate microbial inoculant for forest drought management.

## 1. Introduction

*Pinus sylvestris* var. *mongolica* is an ecologically and economically important geographical variant of *P. sylvestris* native to eastern Asia. It is widely used for reforestation and soil conservation in arid and semi-arid regions of northern China due to its fast growth, strong adaptability, and high resistance to environmental stress [1,2]. As a keystone species in vegetation restoration and desertification control programs, this pine plays a crucial role in maintaining regional ecological stability and preventing soil erosion [3]. However, since the 1990s, *P. sylvestris* var. *mongolica* plantations have experienced growth decline, needle yellowing, and even large scale dieback, which have been increasingly linked to prolonged water limitation [4]. Accordingly, water deficit has become one of the most severe environmental constraints on the survival and productivity of this species [5]. Drought stress disrupts plant physiological homeostasis by causing osmotic imbalance, inhibiting photosynthesis, promoting oxidative damage, and impairing nutrient uptake [6]. Conventional mitigation strategies, such as irrigation optimization, drought tolerant breeding, and genetic modification have shown some success but remain costly, time-consuming, and difficult to implement in large scale forest ecosystems. Consequently, biological approaches using plant growth-promoting rhizobacteria (PGPR) have attracted growing attention as a sustainable, eco-friendly, and cost-effective alternative for enhancing plant drought tolerance [7]. These characteristics make *P. sylvestris* var. *mongolica* an ecologically important but drought-vulnerable afforestation species, and highlight the need for practical strategies to improve its establishment and persistence under increasing water limitation.

PGPR are a group of beneficial soil microorganisms that colonize the rhizosphere and promote plant growth directly or indirectly through multiple mechanisms [8]. Under drought stress, plants commonly experience osmotic imbalance, oxidative damage, reduced photosynthetic efficiency, and impaired nutrient uptake, all of which constrain growth and survival [9]. In this context, PGPR have been reported to enhance plant water use efficiency, regulate osmolyte accumulation, improve antioxidant defense systems, and stimulate the synthesis of phytohormones such as indole-3-acetic acid (IAA). Many PGPR can also produce 1-aminocyclopropane-1-carboxylate (ACC) deaminase, which lowers ethylene levels by degrading its precursor ACC and thereby alleviates stress-induced growth inhibition [10,11]. Additionally, PGPR can improve nutrient availability by solubilizing phosphorus, producing siderophores for iron acquisition, and secreting hydrolytic or redox-active enzymes. Collectively, these traits enable PGPR to reduce drought-induced physiological damage, limit reactive oxygen species (ROS) accumulation, and help maintain photosynthetic performance [12]. Compared with irrigation or breeding-based approaches, PGPR-based regulation is particularly attractive for forest systems because it is low-input, environmentally friendly, and can be applied at the rhizosphere level without intensive engineering management [13].

Previous studies have demonstrated that PGPR inoculation significantly enhances drought tolerance in major crops such as maize [14], wheat [10], and rice [15]. However, the role of PGPR in alleviating drought stress in Pinaceae tree species, particularly *P. sylvestris* var. *mongolica*, remains poorly understood. In addition, studies that simultaneously integrate plant physiological responses, rhizosphere bacterial community variation, soil biochemical changes, and genome-level functional information are still limited in forest tree systems. To address these gaps, this study examined the drought-related responses of *P. sylvestris* var. *mongolica* seedlings to inoculation with *Pseudomonas marginalis* A39 under controlled greenhouse conditions. The experimental framework combined in vitro trait assays, a graded drought pot experiment, rhizosphere bacterial community profiling, genome annotation, and qRT-PCR analysis of selected candidate genes. Rather than testing a single causal pathway, the study aimed to clarify whether A39 inoculation was associated with coordinated changes in seedling physiology, soil biochemical properties, rhizosphere bacterial composition, and genome-inferred functional traits. Specifically, we focused on: (i) evaluating seedling growth, photosynthetic pigments, oxidative stress indicators, antioxidant enzymes, and osmolyte accumulation; (ii) characterizing changes in soil nutrient availability, enzyme activity, and rhizosphere bacterial community composition; and (iii) identifying putative genomic features and drought-responsive expression patterns that may help explain the functional potential of strain A39.

Overall, the novelty of this study lies in the strain-specific and multi-level characterization of *P. marginalis* A39 in a Pinaceae tree species under graded drought stress. Unlike studies that mainly confirm general PGPR drought-alleviating traits, this work integrates plant physiological responses, soil biochemical changes, rhizosphere bacterial community variation, and genome-inferred functional traits to clarify the potential links among A39 inoculation, rhizosphere regulation, and drought-related seedling performance. These findings provide new evidence for understanding PGPR–plant–environment interactions in forest tree systems under water-limited conditions.

## 2. Materials and Methods

### 2.1. Bacterial Strain and Assessment of Drought Tolerance

*Pseudomonas marginalis* A39 was isolated from the rhizosphere soil of *Pinus sylvestris* var. *mongolica* collected in the autumn of 2021 from the Zhanggutai Experimental Forest Farm, Fuxin City, Liaoning Province, China. The 16S rRNA gene sequence of strain A39 has been deposited in GenBank under accession number MT280205. The sampling site is located in a drought-prone sandy plantation region of northeastern China and represents a typical semi-arid afforestation environment where *P. sylvestris* var. *mongolica* frequently experiences seasonal water limitation [16,17]. Strain A39 was selected for further study because it showed superior plant growth-promoting performance, including siderophore production, indole-3-acetic acid (IAA) production, and phosphate-solubilizing ability. The selection of A39 was therefore based on functional screening of culturable rhizobacteria from a drought-prone *P. sylvestris* var. *mongolica* rhizosphere, rather than on prior amplicon-based abundance or network analyses. The strain was identified based on 16S rRNA gene sequencing and phylogenetic analysis, and is currently preserved in the Forest Microbiology Laboratory, Northeast Forestry University, Harbin, China.

Colony morphology of strain A39 was observed on NA plates after incubation at 30 ± 1 °C for 24–48 h. Gram staining was performed using a standard Gram-staining procedure, and cell morphology was observed under a light microscope.

Drought stress was simulated using polyethylene glycol (PEG 6000, 25%, equivalent to −0.73 MPa, CAS No. 25322-68-3, MedChemExpress, Monmouth Junction, NJ, USA), following the method described by Zhang et al. (2020) [14]. This stress level was selected to impose a relatively strong osmotic stress condition for evaluating the drought tolerance and functional stability of strain A39 under controlled in vitro conditions, rather than to directly reproduce field drought conditions. The strain was cultured in nutrient broth (NB) adjusted to −0.73 MPa at 28 ± 3 °C and 200 rpm for 3 days for in vitro drought-tolerance assessment under PEG-induced osmotic stress. Drought tolerance was evaluated by measuring the optical density at 600 nm (OD_600_) using a UV-2355 UV–visible spectrophotometer (UNICO Instrument Co., Ltd., Shanghai, China), which reflected bacterial growth under osmotic stress conditions.

### 2.2. ACC Deaminase Activity and Plant Growth-Promoting Traits

The ACC deaminase activity of strain A39 was determined by measuring the amount of α-ketobutyrate released from ACC deamination, following Carlson and Tugizimana (2020) [18]. Briefly, bacterial cells were collected from fresh culture, washed with sterile saline, and incubated in a reaction system containing ACC as the substrate. After incubation, the reaction was terminated, and α-ketobutyrate production was quantified colorimetrically. ACC deaminase activity was expressed as μmol α-ketobutyrate mg^−1^ protein h^−1^.

PGP traits, including IAA production, siderophore production, and phosphate solubilization, were assayed following Khan and Bano (2019) [19] with minor modifications. For IAA production, strain A39 was cultured in NB supplemented with L-tryptophan, and the culture supernatant was mixed with Salkowski reagent. After color development in the dark, absorbance was measured spectrophotometrically, and IAA concentration was calculated from a standard curve. Siderophore production was determined using the chrome azurol S (CAS) assay, and the result was expressed as siderophore units (SU%) based on the decrease in absorbance of the CAS reagent. Phosphate solubilization was evaluated by culturing A39 in phosphate-solubilizing medium containing insoluble inorganic phosphate as the phosphorus source. After incubation, the culture was centrifuged, and soluble phosphorus in the supernatant was quantified colorimetrically using a standard curve.

All in vitro assays were conducted under two conditions: a control condition without PEG 6000 and a drought-simulating condition with PEG 6000 (25%, equivalent to −0.73 MPa). For each assay, strain A39 was inoculated into the corresponding medium or reaction system under the same initial culture conditions, and three biological replicates were included for each treatment.

### 2.3. Inoculation Procedure and Pot Experiment

A greenhouse pot experiment was conducted at the experimental forest farm of Northeast Forestry University (Harbin, China) from April to October 2023. The growth substrate consisted of peat, vermiculite, and sand at a ratio of 2:1:1 (*v*/*v*/*v*), and was used as a pot cultivation substrate system rather than a natural field-soil system. No sterile-substrate control or microbiome-exclusion treatment was included; therefore, this experiment was designed to evaluate the overall response associated with A39 inoculation in a non-sterile pot system rather than to separate direct bacterial effects from microbiome-mediated effects.

Seeds of *P. sylvestris* var. *mongolica* were purchased from the Zhanggutai Nursery Base, surface-sterilized with 0.5% KMnO_4_ for 15 min, rinsed thoroughly with sterile water, germinated, and sown in 15 × 15 cm plastic pots (20 seeds per pot). The pots were maintained in a greenhouse at 22/30 ± 3 °C (day/night) with a 14 h light/10 h dark photoperiod. After seedling establishment, seedlings were thinned to five uniform individuals per pot before inoculation and drought treatment. Each pot containing five seedlings was considered one biological replicate.

For inoculum preparation, strain A39 was grown in NB at 30 ± 1 °C and 180 rpm for 24 h, harvested by centrifugation, washed, and resuspended to OD_600_ = 0.6, corresponding to approximately 1 × 10^8^ CFU mL^−1^. The bacterial suspension was applied to the rhizosphere at 100 mL per pot at 15-day intervals. On each inoculation day, the bacterial suspension replaced the routine watering volume. Control plants received an equal volume of sterile NB medium to minimize differences in liquid input and medium-derived nutrient input between inoculated and non-inoculated treatments.

Drought stress was initiated 30 days after germination, when seedlings had established uniformly. Four soil relative water content (SRWC) levels, 80%, 60%, 45%, and 30%, were used to represent normal watering (ND), light drought (LD), moderate drought (MD), and severe drought (SD), respectively, following commonly used pot-based drought-gradient designs reported in previous studies [20]. Soil moisture was maintained using a gravimetric method. Briefly, pots were weighed at regular intervals, and water was added according to the amount of water lost to restore each treatment to its target SRWC. This procedure was continued throughout the experimental period to keep the moisture levels as constant as possible.

The experiment included eight treatments: CK_80, CK_60, CK_45, CK_30, A39_80, A39_60, A39_45, and A39_30. Each treatment contained 10 pots as biological replicates, with five seedlings per pot. Thus, a total of 80 pots and 400 seedlings were used in the pot experiment. Soil moisture was maintained daily within the target range using the gravimetric method.

### 2.4. Measurement of Plant and Soil Parameters

After 25 days of drought treatment, plant height, ground diameter, aboveground dry weight (ADW) and underground dry weight (UDW) were measured. Leaf samples were collected for the determination of total chlorophyll (CB10141-Pt), carotenoids (CB10608-Pt), superoxide (O_2_^−^; CB10161-Pt), hydrogen peroxide (H_2_O_2_; CB10046-Pt), proline (PRO; CB10079-Pt), malondialdehyde (MDA; CB10017-Pt), superoxide dismutase (SOD; CB10021-Pt), peroxidase (POD; CB10048-Pt), and catalase (CAT; CB10047-Pt). All indicators were detected using commercial kits (COIBO BIO, Shanghai, China). Briefly, SOD, POD, and CAT were measured by colorimetric assays based on enzyme activity, MDA was determined based on thiobarbituric acid reactive substances, and PRO was measured using a ninhydrin-based colorimetric method. ABA (ABA; product no. H251) and ethylene (ET, product no. RF-Z183; Shanghai Ruifan Biotechnology, Shanghai, China) were quantified using ELISA-based assays. All measurements were performed with three biological replicates.

Soil organic matter (OM), total nitrogen (TN), total phosphorus (TP), total potassium (TK), available nitrogen (AN), available phosphorus (AP), and available potassium (AK) were determined according to the method of Cui et al. (2018) [21] with minor modifications. Briefly, OM was measured using the potassium dichromate oxidation method. TN was determined using the Kjeldahl digestion method. TP and TK were measured after acid digestion, followed by colorimetric determination for TP and flame photometric determination for TK. AN was determined using the alkaline hydrolysis diffusion method. AP was extracted using sodium bicarbonate and quantified colorimetrically, whereas AK was extracted using ammonium acetate and measured by flame photometry.

The activities of soil acid phosphatase (S-Acp, A060), catalase (S-Cat, A007), sucrase (S-Sc, A082), and urease (S-Ue, A121) were measured using commercial kits (Jiancheng Bioengineering Institute, Nanjing, China). These enzymes were selected because they reflect phosphorus transformation, reactive oxygen detoxification, carbon cycling, and nitrogen transformation, respectively, in the rhizosphere under drought stress. Among them, acid phosphatase was included as an indicator of organic phosphorus mineralization and rhizosphere phosphorus activation, rather than as a direct indicator of soil acidity. Briefly, S-Acp activity was determined based on substrate hydrolysis and product color development, S-Cat activity was measured according to the decomposition of hydrogen peroxide, S-Sc activity was evaluated based on the release of reducing sugars, and S-Ue activity was determined according to ammonium production after urea hydrolysis.

### 2.5. Microbial Community and Genomic Analyses

Rhizosphere soil, rather than topsoil or subsoil, was collected for bacterial community analysis. For each treatment, three independent pots were randomly selected as biological replicates. In each selected pot, soil tightly adhering to the roots of five seedlings was collected using a sterile brush and pooled to form one composite rhizosphere soil sample. In total, 24 independent rhizosphere soil samples were used for bacterial community sequencing. The samples were immediately frozen in liquid nitrogen and stored at −80 °C until DNA extraction.

Total microbial DNA was extracted from each rhizosphere soil sample using the E.Z.N.A. Soil DNA Kit (Omega Bio-tek, Norcross, GA, USA) according to the manufacturer’s instructions. DNA concentration and purity were evaluated using a NanoDrop spectrophotometer (Thermo Fisher Scientific, Waltham, MA, USA), and DNA integrity was examined by 1% agarose gel electrophoresis. The V3–V4 hypervariable region of the bacterial 16S rRNA gene was amplified using primers 338F (5′-ACTCCTACGGGAGGCAGCAG-3′) and (806R, 5′-GGACTACHVGGGTWTCTAAT-3′). PCR amplification was performed in triplicate for each sample. The amplification program consisted of initial denaturation at 95 °C for 3 min, followed by 27 cycles of denaturation at 95 °C for 30 s, annealing at 51 °C for 30 s, and extension at 72 °C for 30 s, with a final extension at 72 °C for 10 min.

PCR products were examined by 2% agarose gel electrophoresis and purified using the AxyPrep DNA Gel Extraction Kit (Axygen Biosciences, Union City, CA, USA). The purified amplicons were quantified using a QuantiFluor-ST fluorometer (Promega Corporation, Madison, WI, USA), pooled in equimolar concentrations, and used to construct paired-end amplicon libraries. Sequencing was performed by Majorbio Bio-Pharm Technology Co., Ltd. (Shanghai, China) on an Illumina MiSeq platform using 2 × 300 bp paired-end sequencing chemistry. The amplicon sequencing data were deposited in the NCBI Sequence Read Archive under BioProject accession number PRJNA774501.

Raw paired-end reads were demultiplexed and quality-filtered using fastp (version 0.19.6). Reads were truncated when the average quality score within a 50 bp sliding window was lower than 20. Reads shorter than 50 bp after quality trimming, reads containing ambiguous bases, and reads containing adapter or primer contamination were removed. The remaining paired-end reads were merged using FLASH (version 1.2.11), with a minimum overlap length of 10 bp and a maximum mismatch ratio of 0.20 within the overlapping region. Sequences that could not be successfully merged were discarded.

High-quality sequences were clustered into operational taxonomic units (OTUs) at 97% sequence similarity using UPARSE (version 7.0.1090). Chimeric sequences were identified and removed using UCHIME (version 4.2). A representative sequence from each OTU was selected for taxonomic classification using the RDP Classifier (version 2.11) against the SILVA Release 128 bacterial 16S rRNA gene database, with a confidence threshold of 0.70. Sequences assigned to chloroplasts, mitochondria, archaea, eukaryotes, or non-bacterial lineages were removed. The OTU abundance table was normalized to the minimum number of valid sequences among all samples before community diversity and composition analyses. Rarefaction curves, Shannon diversity indices, OTU distributions, and bacterial relative abundances at the phylum and genus levels were calculated from the normalized OTU table.

Genomic DNA of strain A39 was extracted from fresh bacterial culture and submitted to Shanghai Lingen Biotechnology Co., Ltd. (Shanghai, China) for whole-genome sequencing. Genome sequencing combined Illumina short-read sequencing and PacBio single-molecule real-time long-read sequencing. Illumina reads were quality-filtered using fastp, whereas PacBio reads were processed using the SMRT Analysis pipeline version 2.3.0. Short-read assembly was performed using SOAPdenovo (version 2.0), and the hybrid genome assembly was generated using Unicycler (version 0.10). Transfer RNA and ribosomal RNA genes were identified using tRNAscan-SE (version 1.31) and Barrnap (version 0.4.2), respectively. Predicted gene functions were annotated against the Gene Ontology, Cluster of Orthologous Groups/eggNOG, and Kyoto Encyclopedia of Genes and Genomes databases. Genome assembly and annotation quality were summarized using scaffold number, scaffold N50, estimated sequencing coverage, GC content, predicted gene number, non-coding RNA genes, and functional annotation statistics.

The genome-based taxonomic affiliation of strain A39 was evaluated using phylogenomic and genome-similarity analyses. The assembled draft genome was analyzed using the Type (Strain) Genome Server (TYGS) to obtain digital DNA–DNA hybridization values. Average nucleotide identity based on BLAST (version 2.2.29) was calculated between strain A39 and the closely related type strains *Pseudomonas marginalis* ICMP 3553 and *Pseudomonas petroselini* MAFF 311094. Species-level thresholds of approximately 95–96% ANI and 70% dDDH were used as reference criteria for taxonomic interpretation. The ANI and dDDH results are presented in Supplementary Table S1.

### 2.6. qRT-PCR Analysis of Selected Functional Genes Under Different Drought Gradients

Based on genome annotation, eight genes putatively associated with drought adaptation or plant growth-promoting traits were selected for qRT-PCR analysis. These genes were used as candidate functional markers to evaluate the transcriptional response of strain A39 under different drought gradients; they were not considered experimentally validated determinants of plant drought tolerance. Total RNA was extracted from the harvested cell pellets using the Bead/SDS/phenol method. Specifically, for each treatment, 5 mL of bacterial culture was processed, and the resulting cell pellet was immediately used for RNA isolation. The extracted RNA was then reverse-transcribed into complementary DNA (cDNA) using the RevertAid First Strand cDNA Synthesis Kit (Thermo Fisher Scientific, Waltham, MA, USA). qRT-PCR was performed on a Rotor-Gene 6000 instrument (QIAGEN, Hilden, Germany) using SYBR Premix Ex Taq chemistry in a 25 μL reaction system. Each 25 μL reaction mixture contained SYBR Premix Ex Taq (RR420A, TaKaRa Bio Inc., Kusatsu, Shiga, Japan), ROX reference dye, forward and reverse primers at a final concentration of 0.2 μM each, and 1 μL of cDNA template. gyrB was used as the internal reference gene. Relative expression levels were calculated using the 2^−ΔΔCt^ method. The analyzed genes included *pyk* (pyruvate kinase), *nifU* (nitrogen-fixation protein), *SOD2* (superoxide dismutase), *catB* (catalase), *acdS* (1-aminocyclopropane-1-carboxylate deaminase), *kdpA* (potassium-transporting ATPase potassium-binding subunit), *putA* (proline dehydrogenase), and *iaaM* (IAA monooxygenase). Primer sequences for all tested genes are listed in Supplementary Table S2.

### 2.7. Data and Statistical Analyses

All experiments were arranged in a completely randomized design with at least three biological replicates. Before analysis of variance (ANOVA), data normality and homogeneity of variances were checked using the Shapiro–Wilk test and Levene’s test, respectively. Data from the pot experiment were analyzed by two-way ANOVA, with inoculation treatment and drought level as fixed factors, followed by Duncan’s multiple range test for post hoc comparison of treatment means at *p* < 0.05. Data from the in vitro drought-tolerance and plant growth-promoting trait assays were analyzed by one-way ANOVA or Student’s *t*-test, as appropriate. Pearson correlation analysis was used to evaluate relationships between plant physiological traits and soil biochemical properties. For key plant and soil variables, treatment effects were also expressed as percentage changes relative to the corresponding non-inoculated controls, and coefficients of variation (CV, %) were calculated to describe data dispersion and support biological interpretation.

Alpha diversity of rhizosphere bacterial communities was evaluated using the Shannon index, and rarefaction curves were generated from the rarefied OTU abundance table. Beta diversity was assessed using principal coordinate analysis (PCoA) based on Bray–Curtis dissimilarity matrices at the genus level. Permutational multivariate analysis of variance (PERMANOVA) with 999 permutations was performed to test differences in bacterial community composition among the eight treatments. Differentially enriched bacterial taxa were identified using linear discriminant analysis effect size (LEfSe), with statistical significance defined as *p* < 0.05 and an LDA score > 4.0. Redundancy analysis (RDA) and Mantel tests were used to evaluate the relationships between bacterial community composition and soil environmental variables. Bacterial co-occurrence networks were constructed based on Spearman correlations among genera meeting the specified abundance and occurrence criteria. Within-module connectivity (Zi) and among-module connectivity (Pi) were calculated to determine the topological roles of bacterial taxa. Statistical analyses were performed using SPSS 25.0 (SPSS Inc., Chicago, IL, USA), and figures were prepared using OriginPro 9.0.

## 3. Results

### 3.1. Drought Tolerance and PGP Traits of Strain A39

Strain A39 formed creamy-white, round, and smooth colonies on NA medium and showed rod-shaped cells with Gram-negative staining characteristics (Supplementary Figure S1). Under drought-mimicking conditions (−0.73 MPa), strain A39 maintained substantial growth, although its OD_600_ value was significantly lower than that under non-stress conditions (*p* < 0.05; Table 1). Despite osmotic stress, A39 retained multiple PGP traits, including the production of IAA, ACC deaminase, siderophores, and phosphate solubilization, although the magnitudes of these traits varied under drought stress. IAA synthesis showed no significant difference between drought and control treatments while siderophore production, ACC deaminase activity, and phosphate solubilization were significantly reduced (*p* < 0.05).

### 3.2. Effects of A39 Inoculation on Plant Biomass, Soil Nutrients, and Enzymatic Activities

#### 3.2.1. Plant Growth and Biomass Accumulation

**Figure 1:**
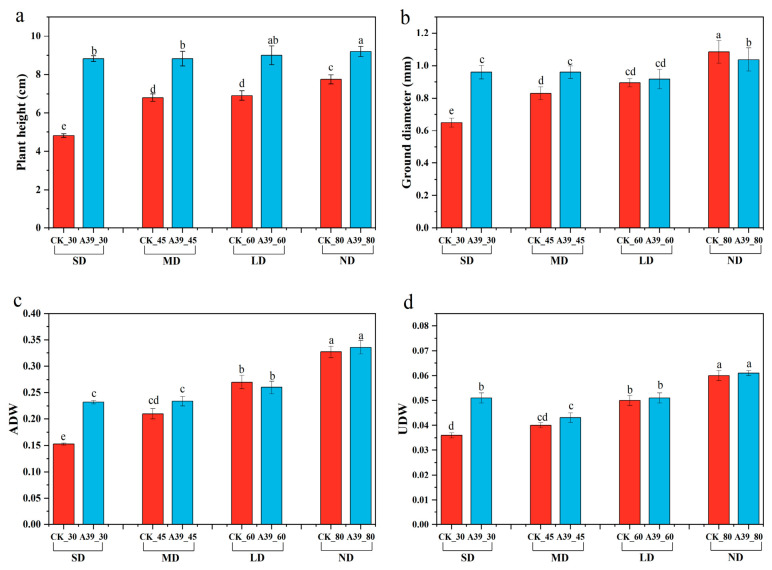
Effects of strain A39 inoculation on plant growth and dry biomass accumulation of *Pinus sylvestris* var. *mongolica* under diverse drought gradients. (**a**) Plant height. (**b**) Ground diameter. (**c**) Aboveground dry weight (ADW). (**d**) Underground dry weight (UDW). Data are presented as mean ± standard deviation (*n* = 10). CK: Uninoculated control; A39: A39-inoculated treatment. Data are presented as mean ± standard deviation (*n* = 10 for growth parameters). Two-way ANOVA was performed with inoculation treatment and drought level as fixed factors, followed by Duncan’s multiple range test at *p* < 0.05. Lowercase letters indicate significant differences among CK and A39 treatments under the same drought gradient.

Two-way ANOVA indicated significant effects of drought level and inoculation treatment on plant growth, biomass accumulation, with significant interaction effects observed for some variables (Figure 1a–d). A39 inoculation significantly mitigated this inhibition, with the degree of mitigation varying across drought levels. Specifically, under all drought gradients, inoculation with A39 significantly increased the seedling height, with an increase range of 18.71% (ND) to 83.58% (SD) compared with the non-inoculated control (*p* < 0.05; Figure 1a). Only under SD stress, inoculated seedlings exhibited higher ground diameter, ADW and UDW. Compared with the SD control, the above parameters increased by 47.69%, 37.14% and 41.67%, respectively (*p* < 0.05; *n* = 10; Figure 1b–d).

#### 3.2.2. Soil Nutrient Contents and Enzymatic Activities

**Figure 2:**
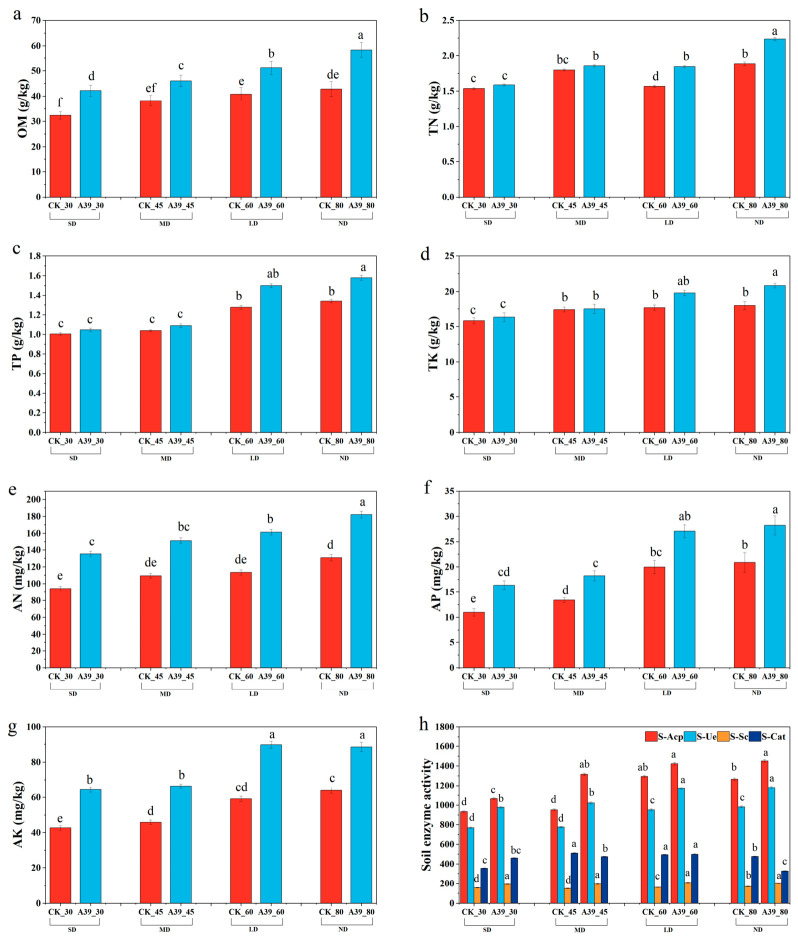
Effects of strain A39 inoculation on soil nutrient contents and enzyme activities in the rhizosphere of *Pinus sylvestris* var. *mongolica* under diverse drought gradients. (**a**) Organic matter (OM) content. (**b**) Total nitrogen (TN) content. (**c**) Total phosphorus (TP) content. (**d**) Total potassium (TK) content. (**e**) Available nitrogen (AN) content. (**f**) Available phosphorus (AP) content. (**g**) Available potassium (AK) content. (**h**) Soil enzyme activities, including acid phosphatase (S-Acp), urease (S-Ue), sucrase (S-Sc), and catalase (S-Cat). Data are presented as mean ± standard deviation (*n* = 3). Two-way ANOVA was performed with inoculation treatment and drought level as fixed factors, followed by Duncan’s multiple range test at *p* < 0.05. Lowercase letters indicate significant differences between CK and A39 treatments under the same drought gradient.

A39 inoculation enhanced soil nutrient availability under all drought intensities (Figure 2a–g). The OM content was significantly higher in inoculated soils, particularly under ND stress, where the increase reached 36.13% (Figure 2a). Although TN, TP, and TK contents were not significantly affected at MD and SD stress (Figure 2b–d), available nutrients (AN, AP, AK) increased consistently across all treatments by more than 35% (*p* < 0.05; Figure 2e–g).

A39 inoculation significantly enhanced the activities of S-Acp, S-Sc, and S-Ue under all drought levels, with maximum increases observed under MD stress (38.29%, 32.15%, and 31.75%, respectively; *p* < 0.05, Figure 2h). In contrast, S-Cat activity responded significantly only under SD conditions, increasing by 29.65% relative to control (*p* < 0.05; Figure 2h).

### 3.3. Effects of A39 Inoculation on Photosynthetic Pigments, ROS Accumulation, Membrane Lipid Peroxidation, and Antioxidant Enzyme Activities

#### 3.3.1. Photosynthetic Pigments

**Figure 3:**
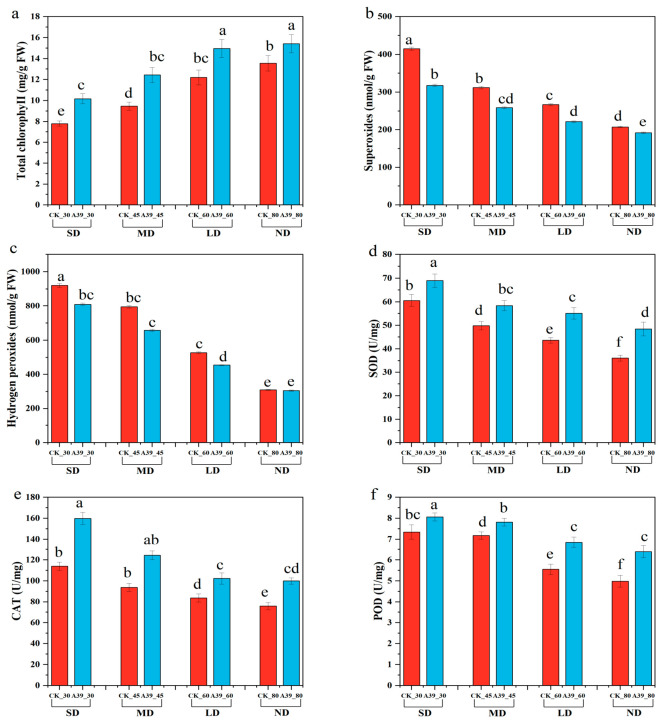
Effects of strain A39 inoculation on photosynthetic pigment status, ROS accumulation, and antioxidant enzyme activities of *Pinus sylvestris* var. *mongolica* under diverse drought gradients. (**a**) Total chlorophyll content. (**b**) Superoxide anion (O_2_^−^) content. (**c**) Hydrogen peroxide (H_2_O_2_) content. (**d**) Superoxide dismutase (SOD) activity. (**e**) Peroxidase (POD) activity. (**f**) Catalase (CAT) activity. Data are presented as mean ± standard deviation (*n* = 3). Two-way ANOVA was performed with inoculation treatment and drought level as fixed factors, followed by Duncan’s multiple range test at *p* < 0.05. Lowercase letters indicate significant differences among CK and A39 treatments under the same drought gradient.

Drought stress significantly reduced total chlorophyll and carotenoid contents (*p* < 0.05), whereas A39 inoculation partially alleviated these decreases. Under MD and SD stress, total chlorophyll increased by 31.22% and 30.38%, respectively, compared with uninoculated controls (*p* < 0.05; Figure 3a). Carotenoids content showed a similar pattern and increased by 109.40% and 118.37% under MD and SD, respectively (*p* < 0.05; Supplementary Figure S2a).

#### 3.3.2. ROS Accumulation and Membrane Lipid Peroxidation

Drought stress induced significant accumulation of O_2_^−^ and H_2_O_2_ in seedling leaves (Figure 3b,c). Inoculation with A39 effectively reduced ROS accumulation under all drought levels. Compared with the ND control, the O_2_^−^ production of non-inoculated seedlings increased by 28.99%, 50.72%, and 100.48% under LD, MD, and SD stresses, respectively, whereas these increases were reduced to 32.18%, 36.24%, and 44.10% after A39 inoculation (*p* < 0.05). Similarly, the H_2_O_2_ production of non-inoculated seedlings increased by 70.32%, 156.45%, and 197.10% under LD, MD, SD stress, respectively, while the increase was only 49.15% (LD), 42.47% (MD), 29.72% (SD) after inoculation with strain A39 (*p* < 0.05).

The contents of MDA and PRO both increased significantly under drought stress (*p* < 0.05; Supplementary Figure S2b,c). Under ND and LD stresses, inoculation with strain A39 had no significant effect on the contents of MDA and PRO. However, under MD and SD stresses, inoculation with strain A39 significantly reduced the contents of both substances: compared with the control, the MDA content decreased by 43.01% and 33.71% under MD and SD, respectively, and the PRO content decreased by 53.16% and 35.40% under MD and SD, respectively (*p* < 0.05).

#### 3.3.3. Antioxidant Enzyme Activities

SOD, POD, and CAT activities increased significantly with drought intensity (Figure 3d–f). Inoculation with strain A39 further enhanced the activities of these enzymes, but the optimal drought gradients varied among different enzymes: the increments in SOD and POD activities were the highest under the ND gradient, reaching 34.21% and 28.22%, respectively (*p* < 0.05); the increment in CAT activity was the highest under the SD gradient, increasing by 40.20% compared with the control (*p* < 0.05).

### 3.4. Correlation Between Plant Physiological Parameters and Soil Biochemical Properties

Pearson correlation analysis revealed significant correlations between plant parameters and soil parameters (Table 2). The specific results were as follows: ROS (including O_2_^−^ and H_2_O_2_) and membrane lipid peroxidation products (MDA and PRO) showed extremely significant negative correlations with photosynthetic pigments (*p* < 0.01) and significant negative correlations with soil properties (*p* < 0.05), but no significant correlations with growth traits or biochemical traits; Soil nutrients (OM, TN, TP, TK, AN, AP, and AK) and enzyme activities (S-Acp, S-Cat, S-Sc, and S-Ue) exhibited extremely significant positive correlations with photosynthetic pigments (*p* < 0.01), but no significant correlations with biochemical traits; Soil OM, AN, AK, S-Ue, and S-Sc showed significant positive correlations with growth traits (*p* < 0.05), while TK displayed an extremely significant positive correlation with growth traits (*p* < 0.01).

### 3.5. Effects of Inoculation with Strain A39 on Rhizosphere Bacterial Community Diversity and Composition

**Figure 4:**
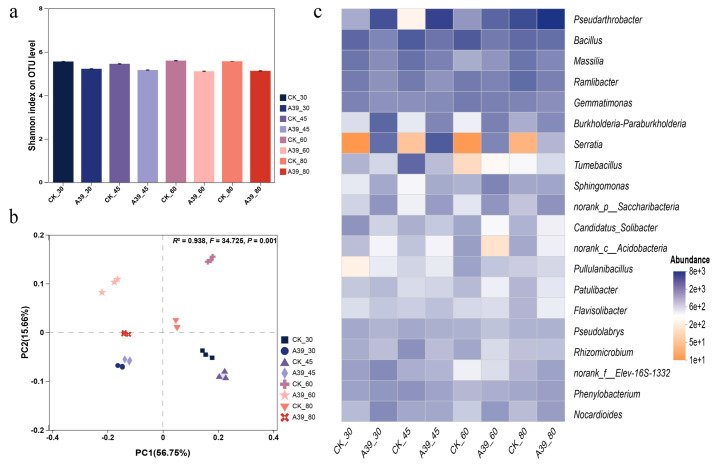
Effects of A39 inoculation on rhizosphere bacterial communities under different drought gradients. (**a**) Shannon index. (**b**) PCoA of bacterial community structure. (**c**) Heatmap of dominant bacterial genera. CK and A39 indicate non-inoculated and A39-inoculated treatments, respectively; 30, 45, 60, and 80 correspond to SD, MD, LD, and ND, respectively. PCoA was performed based on Bray–Curtis distances at the genus level. PERMANOVA with 999 permutations confirmed significant differences in bacterial community composition among the eight treatments (*R*^2^ = 0.938, *F* = 34.725, *p* = 0.001).

Alpha-diversity analysis showed that the Shannon index of rhizosphere bacterial communities was generally lower in the A39-inoculated treatments than in the corresponding non-inoculated controls across different drought gradients (Figure 4a), indicating that A39 inoculation was associated with changes in bacterial alpha diversity and community evenness. PCoA based on Bray–Curtis distances at the genus level showed clear separation of rhizosphere bacterial communities among the eight treatments (Figure 4b). PERMANOVA further confirmed significant differences in bacterial community composition among treatments (*R*^2^ = 0.938, *F* = 34.725, *p* = 0.001), indicating that drought level and A39 inoculation jointly contributed to rhizosphere bacterial community variation. At the genus level, the dominant taxa included *Pseudarthrobacter*, *Bacillus*, *Massilia*, *Ramlibacter*, and *Gemmatimonas*, and their abundance patterns varied among drought gradients and inoculation treatments (Figure 4c).

At the phylum level, the rhizosphere bacterial community was mainly composed of Proteobacteria, Actinobacteria, Firmicutes, and Acidobacteria, with Proteobacteria remaining the dominant phylum across all treatments (Supplementary Figure S5a). Compared with the corresponding controls, A39 inoculation generally increased the relative abundance of Actinobacteria and altered the relative abundances of several other dominant phyla under drought conditions. Linear discriminant analysis effect size (LEfSe) analysis further identified multiple bacterial taxa with significantly different relative abundances among treatments, indicating that A39 inoculation, together with drought stress, was associated with shifts in rhizosphere bacterial community composition (Supplementary Figure S5b). Rarefaction curves and OTU distribution among treatments are shown in Supplementary Figure S3 and Figure S4, respectively.

### 3.6. Key Taxa and Environmental Drivers

**Figure 5:**
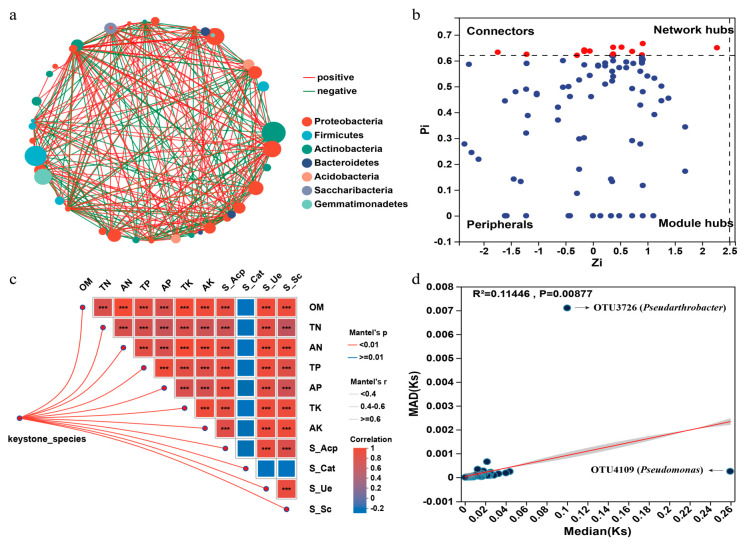
Key taxa identification and their correlation with soil environmental parameters in the rhizosphere microbial community. (**a**) Rhizosphere microbial co-occurrence network of key taxa. (**b**) Zi-Pi plot for core genus identification. (**c**) Mantel test for correlation between key taxa and soil environmental parameters. (**d**) Keystone species analysis of rhizosphere microbiota. The co-occurrence network was constructed based on correlation method and thresholds. In the Zi–Pi plot (**b**), red dots represent connectors (Pi > 0.62), whereas blue dots represent peripheral taxa (Pi ≤ 0.62 and Zi ≤ 2.5). In panel (**c**), *** indicates a significant correlation at *p* < 0.001.

Co-occurrence network analysis identified 45 genera across six bacterial phyla as key taxa in the rhizosphere community (Figure 5a), and 13 core genera were further confirmed via Zi-Pi analysis (Figure 5b). A Mantel test revealed a significant correlation between the composition of these key taxa and multiple soil environmental parameters (*p* < 0.05, Figure 5c). Keystone species analysis revealed that OTU3726 (*Pseudarthrobacter*) and OTU4109 (*Pseudomonas*) were critical keystone species (Figure 5d).

**Figure 6:**
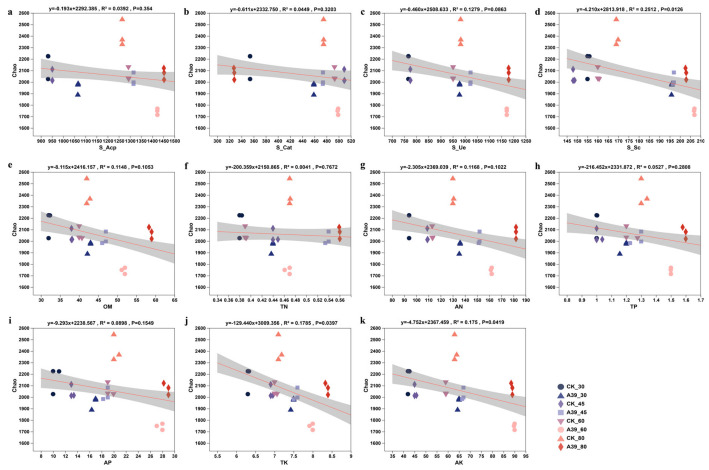
Sorting regression analysis between rhizosphere key genera and soil environmental drivers. (**a**) Soil acid phosphatase (S-Acp); (**b**) soil catalase (S-Cat); (**c**) soil urease (S-Ue); (**d**) soil sucrase (S-Sc); (**e**) organic matter (OM); (**f**) total nitrogen (TN); (**g**) available nitrogen (AN); (**h**) total phosphorus (TP); (**i**) available phosphorus (AP); (**j**) total potassium (TK); (**k**) available potassium (AK).

Sorting regression analysis indicated that, among 11 measured soil variables, S-Sc, AK and TK had significant effects on microbial community richness (*p* < 0.05). Notably, richness declined monotonically with increasing values of these three variables (Figure 6).

### 3.7. Genome-Based Taxonomic Assignment, Genomic Features, and Putative Functional Genes Related to Plant Growth Promotion and Drought Adaptation

Genome-level taxonomic analysis further clarified the species assignment of strain A39. TYGS analysis assigned A39 to *Pseudomonas marginalis*. The dDDH values between A39 and the closely related type strains *P. marginalis* ICMP 3553 and *P. petroselini* MAFF 311094 were 86.5% and 86.0%, respectively. ANIb analysis showed values of 97.54% with *P. marginalis* ICMP 3553 and 97.53% with *P. petroselini* MAFF 311094. Based on the TYGS species assignment together with the ANI and dDDH results, strain A39 was designated as *Pseudomonas marginalis* A39 in this study (Supplementary Table S1).

The draft genome assembly of strain A39 was 6,834,719 bp in length and contained 149 scaffolds, with a scaffold N50 of 114,811 bp and an estimated sequencing coverage of 173.8×. The GC content was 60.5%, and the N rate was 0.00017. Genome annotation predicted 6190 genes, including 6007 protein-coding genes, 57 tRNA genes, and three rRNA genes. Functional annotation assigned 5278 genes to the COG/eggNOG database, 1517 genes to the GO database, and 3502 genes to the KEGG database. These assembly and annotation statistics indicate that the genome data were suitable for downstream comparative and functional annotation analyses (Supplementary Table S3). The genome map and the distributions of genes assigned to COG/eggNOG, GO, and KEGG functional categories are shown in Supplementary Figures S6–S9.

Genome annotation identified a set of putative functional genes that may be related to the plant growth-promoting potential and drought adaptation of strain A39. Representative genes are summarized in Table 3, and the complete annotation list is provided in Supplementary Table S4. These genes were assigned to functional categories associated with related to nitrogen metabolism, carbon metabolism linked to phosphate mobilization, potassium transport, IAA-related metabolism, iron acquisition, oxidative stress defense, ACC deaminase activity, and osmotic adaptation. Because these assignments were based on sequence annotation, they should be interpreted as genome-inferred functional potential rather than direct evidence of gene-level mechanisms in plant drought tolerance.

### 3.8. Relative Expression of Selected Candidate Genes Under Different Drought Gradients

**Figure 7:**
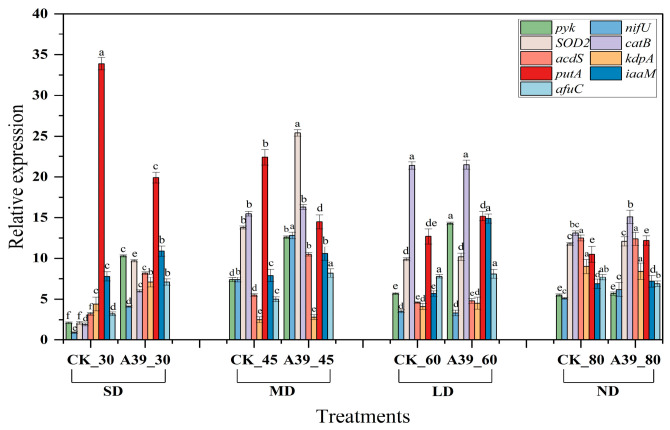
Relative expression of selected functional genes under different drought gradients. Different lowercase letters indicate significant differences among treatments for the same gene at *p* < 0.05.

The relative expression patterns of selected functional genes under different drought gradients are shown in Figure 7. Under ND conditions, no significant differences were observed in the expression levels of the detected genes between CK and A39 treatments. Under LD conditions, only *pyk* and *iaaM* were significantly upregulated in the A39 treatment, showing 1.51- and 1.61-fold increases, respectively, compared with the control (*p* < 0.05), whereas the other genes showed no significant changes. Under MD conditions, in addition to *pyk* and *iaaM*, *nifU*, *SOD2*, and *kdpA* were also significantly upregulated (*p* < 0.05). Under SD conditions, all detected genes except *putA* showed significantly increased expression in the A39 treatment (*p* < 0.05), and *pyk* exhibited the greatest increase, reaching 3.90-fold, whereas *putA* decreased significantly to 0.70-fold of the control. The upregulation of genes related to oxidative stress defense, potassium transport, IAA-related metabolism, ACC deaminase activity, and nutrient-associated metabolism was broadly consistent with the enhanced antioxidant responses, reduced oxidative damage, improved seedling growth, and increased soil nutrient availability observed in A39-inoculated treatments. The expression patterns of these candidate genes indicate that A39 altered its transcriptional activity in response to water limitation. However, these data should be interpreted as expression-level evidence for putative functional markers, not as direct proof that the tested genes causally determine the drought response of the host plant. The upregulation of genes related to oxidative stress defense, potassium transport, IAA-related metabolism, ACC deaminase activity, iron acquisition, and nutrient-associated metabolism was broadly consistent with the enhanced antioxidant responses, reduced oxidative damage, retained PGP traits, improved seedling growth, and increased soil nutrient availability observed in A39-inoculated treatments. CV values for the main measured variables are provided in Supplementary Table S5.

## 4. Discussion

Strain A39 also maintained measurable growth and several plant growth-promoting traits under PEG-induced osmotic stress, consistent with reports that *Pseudomonas fluorescens* and *Pseudomonas bijieensis* can retain growth activity under similar water-potential conditions.

This study evaluated plant physiological, soil biochemical, rhizosphere microbial, and genome-level responses associated with *P. marginalis* A39 inoculation under graded drought stress in *P. sylvestris* var. *mongolica*. Compared with non-inoculated controls, A39-inoculated seedlings showed better growth performance, lower oxidative damage, stronger antioxidant enzyme responses, improved rhizosphere biochemical status, and distinct bacterial community patterns under drought conditions. These responses suggest that A39 inoculation may contribute to drought performance through coordinated plant–soil–microbiome changes, although the underlying causal pathways remain to be experimentally verified. This interpretation is consistent with previous studies showing that beneficial rhizosphere microorganisms can enhance plant performance under water-limited conditions by improving rhizosphere functioning and stress-related physiological responses [22]. In the present study, the positive responses associated with A39 inoculation were more evident under MD and SD conditions, indicating that the effect of A39 was particularly apparent under moderate to severe water limitation. Strain A39 also maintained measurable growth and several plant growth-promoting traits under PEG-induced osmotic stress, consistent with reports that *Pseudomonas fluorescens* and *Pseudomonas bijieensis* can retain growth activity under similar water-potential conditions [23]. Furthermore, A39 maintained relatively high levels of IAA synthesis and phosphate solubilization under drought conditions, which may have contributed to improved nutrient acquisition and plant performance, in agreement with previous reports on the role of *Pseudomonas* spp. in improving drought tolerance through IAA and phosphate solubilization pathways [24,25]. Because no sterile control was included in the present experiment, the contribution of bacterial phosphate-solubilization-related activity to the observed drought-alleviating effect should be interpreted with caution. Although control plants received an equal volume of sterile NB medium, the contribution of bacterial cells and their associated metabolites could not be fully separated from the overall inoculation treatment under the present experimental system. Therefore, the observed plant, soil, and rhizosphere responses should be interpreted as the net effect of A39 inoculation within a non-sterile cultivation context. In addition, root architecture, water uptake efficiency, and hydraulic conductivity were not measured in the present study; therefore, the contribution of belowground water-acquisition traits to the improved drought performance of A39-inoculated seedlings remains unresolved. While there was a slight reduction in the activity of iron-chelating and ACC deaminase under drought stress, these activities were still maintained at significant levels. ACC deaminase plays a crucial role in breaking down the accumulated ACC in plants, reducing ethylene production, and facilitating iron uptake, thus alleviating physiological damage caused by drought [26,27,28]. These findings corroborate those of Rashid et al. (2022) [29], who demonstrated that PGPR strains alleviated drought stress in wheat by maintaining IAA and ACC deaminase activity. Taken together, these results suggest that A39-associated drought response was not driven by a single trait. Instead, maintained plant growth-promoting traits and improved rhizosphere conditions likely worked together with enhanced antioxidant defense to support the overall drought performance of *P. sylvestris* var. *mongolica*.

In terms of physiological responses, A39-treated plants exhibited increased chlorophyll and carotenoid content, while ROS levels and MDA, significantly decreased, indicating its role in maintaining the stability of photosynthetic systems and cellular membrane integrity [30] (Figure 3). The lower PRO accumulation in A39-inoculated seedlings may reflect reduced drought stress intensity rather than loss of osmotic protection [31]. In parallel, higher SOD, POD, and CAT activities, together with lower ROS and MDA levels, indicate that A39 inoculation was associated with stronger antioxidant protection [32]. The enzyme-specific response differed among drought levels: SOD and POD were more responsive under ND conditions, whereas CAT showed the strongest increase under SD stress, partly consistent with previous PGPR studies [33,34]. This pattern may reflect the greater role of CAT in H_2_O_2_ removal under severe oxidative pressure [35,36].

From a soil microenvironment perspective, A39 improved soil OM and available nutrient contents, and several key soil enzyme activities, thereby creating more favorable conditions for the growth of *P. sylvestris* var. *mongolica* under drought stress (Figure 2). This effect was particularly evident under drought conditions, where A39 inoculation increased OM and nutrient availability and enhanced the activities of S-Acp, S-Sc, and S-Ue. This pattern is consistent with previous studies showing that soil enzymes play important roles in nutrient cycling, with S-Sc contributing to carbon utilization and S-Acp promoting organic phosphorus mineralization [37]. Correlation analysis further supported close links between these soil biochemical properties and plant growth traits (Table 2), suggesting that improvement in the soil microenvironment is an important pathway by which A39 promotes plant growth and alleviates drought stress [38,39].

In this study, A39 inoculation was associated with a lower Shannon index across drought gradients, indicating changes in both bacterial diversity and community evenness (Figure 4). This pattern may reflect selective enrichment of particular rhizosphere taxa under altered nutrient conditions, especially because A39 inoculation increased several soil nutrient indicators. Previous studies have shown that shifts in nitrogen and phosphorus availability can reduce bacterial diversity under certain conditions [40]. However, this interpretation remains tentative because nutrient thresholds were not directly tested in the present study. Importantly, lower diversity should not be regarded as uniformly beneficial, as it may also indicate reduced community redundancy and potentially weaker long-term stability of the rhizosphere microbiome [41]. Therefore, the decrease in Shannon diversity was interpreted as a trade-off between selective community restructuring and a possible loss of microbial evenness or resilience. In terms of bacterial community composition, Proteobacteria remained the dominant phylum across all samples (Supplementary Figure S5), consistent with previous studies [42,43]. However, unlike most reports that show a decrease in Proteobacteria abundance under drought stress, with concurrent increases in Actinobacteria and Firmicutes [44], Proteobacteria remained dominant in this study. This discrepancy may arise from the unique metabolic characteristics of A39 and its rhizosphere improvement effects.

Following A39 inoculation under varying drought intensities, significant changes in the rhizosphere bacterial community composition were observed, with *Pseudarthrobacter* and *Bacillus* being the dominant genera (Figure 4). LEfSe analysis further revealed that 67 bacterial taxa showed significantly different relative abundances across the 8 treatments (LDA > 4, *p* < 0.05), suggesting that A39 inoculation, in combination with drought stress, significantly affected the rhizosphere microbial community structure. These compositional changes may be ecologically relevant because several dominant or keystone genera have been linked to drought adaptation and nutrient-related functions. *Bacillus* is frequently reported as a drought-tolerant PGPR genus with ACC deaminase activity, IAA production, and siderophore-related traits [45,46]. *Pseudarthrobacter*, a drought- and nutrient-poor-soil-associated actinobacterial genus, has been associated with organic acid metabolism and phosphate mobilization [47,48], which is consistent with the increased available phosphorus and other nutrient indicators observed in A39-inoculated soils. In addition, *Pseudomonas* species are often recognized as important rhizosphere members involved in organic matter turnover, community stability, and plant stress adaptation [49,50,51]. A39 was selected through functional screening of culturable rhizobacteria, rather than by prior abundance or keystone-taxon analysis. Therefore, the microbial community data provide ecological context for the A39-associated rhizosphere response, but do not directly demonstrate A39 colonization or strain-level abundance changes. Therefore, the shifts in these taxa suggest that A39 inoculation was associated not only with changes in bacterial composition, but also with potential functional reorganization of the rhizosphere community. Mantel test analysis confirmed significant correlations between key microbial genera and soil environmental factors (*p* < 0.05, Figure 5). Notably, this study identified S-Sc, AK, and TK as key environmental factors influencing rhizosphere microbial community structure (Figure 6), thus contributing new insights into the environmental drivers of PGPR-mediated microbial regulation in soil. However, these microbial responses should be interpreted within the context of the present greenhouse pot system. Because the experiment used an artificial substrate and did not include sterile-substrate or microbiome-exclusion controls, the observed community shifts cannot be attributed solely to direct A39–plant interactions. Rhizosphere outcomes may also involve interactions among resident microorganisms, including competition, cooperation, adhesion, and biofilm-related processes [52,53]. Therefore, future studies using colonization assays, microbiome-reduced systems, and field trials are needed to clarify the persistence of A39 and to distinguish direct bacterial effects from microbiome-mediated pathways.

At the molecular level, genome annotation of A39 identified candidate genes putatively related to plant growth-promoting traits and drought adaptation, including genes involved in nitrogen metabolism, carbon metabolism associated with phosphate mobilization, potassium transport, IAA-related metabolism, ACC deaminase activity, and iron acquisition (Table 3; Supplementary Table S3). These annotations provide genome-level clues to the possible functional capacity of A39 under drought conditions, but they should not be regarded as direct evidence of confirmed mechanisms [54]. qRT-PCR analysis further showed that the expression of selected candidate genes varied across drought gradients (Figure 7). Under severe drought, *nifU*, *pyk*, *kdpA*, *afuC*, *iaaM*, *acdS*, *SOD2*, and *catB* were upregulated, whereas *putA* was downregulated. The upregulation of *SOD2* and *catB* was consistent with the enhanced antioxidant enzyme activities and reduced ROS and MDA accumulation observed in A39-inoculated seedlings. The increased expression of *iaaM* and *acdS* corresponded to the retained IAA production and ACC deaminase activity of A39 under osmotic stress, while *afuC* was associated with the siderophore-related iron acquisition potential of this strain. In addition, the upregulation of *kdpA* may reflect a potential role of potassium transport in bacterial osmotic adaptation, and the changes in *nifU* and *pyk* may be related to nutrient- and energy-metabolism responses under drought stress. Similar stress-responsive expression patterns of functional genes have also been reported in beneficial bacteria under adverse conditions [55]. However, these relationships remain inferential because they are based on genome annotation and expression patterns rather than targeted functional validation. Therefore, the tested genes should be interpreted as candidate contributors to the A39-associated drought response, rather than experimentally validated determinants of plant drought tolerance [56,57].

Root colonization and persistence are also important for the performance of PGPR under drought stress. Previous studies have shown that reduced root colonization may weaken the growth-promoting effect of some PGPR strains under water-limited conditions [58]. In the present study, A39 colonization was not directly quantified by re-isolation, strain-specific qPCR, or marker-assisted colonization assays. Therefore, the observed plant, soil, and rhizosphere responses should be interpreted as A39-associated effects rather than direct evidence of root colonization or strain persistence. The upregulation of *kdpA* under severe drought may indicate a potential role of potassium transport in the osmotic adaptation of A39, which could help maintain bacterial activity under water-limited conditions [59,60], but this interpretation remains inferential and requires direct physiological and genetic validation. Overall, the main contribution of this study is the strain-specific integration of osmotic-stress tolerance, retained PGP traits, plant physiological responses, rhizosphere biochemical changes, microbial-community shifts, and genome-inferred functional potential of A39 in a forest-tree system. Compared with previous studies on *Pseudomonas*-mediated drought tolerance mainly conducted in crops or short-term model systems, the present study extends this framework to *P. sylvestris* var. *mongolica*, a conifer species widely used in afforestation in drought-prone sandy regions. The stronger A39-associated responses under MD and SD conditions suggest that this strain may be more relevant under moderate to severe water limitation than under non-stressed conditions. However, because this study was conducted in a controlled greenhouse pot system using an artificial substrate and a relatively short drought-treatment period, the present findings should not be directly extrapolated to field conditions. Before A39 can be developed as a microbial inoculant for forestry, several practical issues need to be addressed, including suitable formulation and carrier materials, survival during storage and nursery application, environmental adaptability in drought-prone sandy soils, biosafety, long-term persistence in the rhizosphere, and compatibility with large-scale seedling production and afforestation practices. In addition, the relatively small number of biological replicates for soil biochemical analyses and microbial community sequencing may limit the statistical robustness of these datasets; therefore, these results were interpreted together with effect magnitudes, consistency across drought gradients, and coherence with plant physiological responses. Future studies involving colonization assays, targeted gene functional validation, long-term field trials, formulation optimization, and biosafety assessment are still required to confirm the ecological stability and application potential of A39 as a candidate PGPR resource for forest seedlings under drought-prone conditions.

## 5. Conclusions

**Figure 8:**
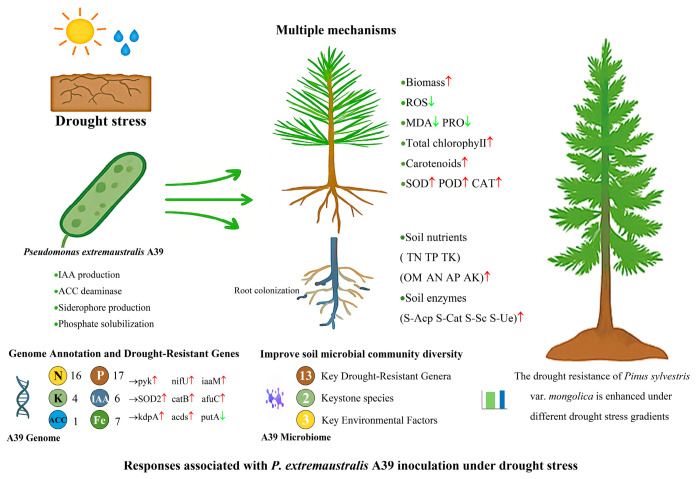
Summary diagram of the responses associated with strain A39 inoculation in *Pinus sylvestris* var. *mongolica* under drought stress. Upward arrows indicate increased responses, and downward arrows indicate decreased responses. ROS, reactive oxygen species; MDA, malondialdehyde; PRO, proline; SOD, superoxide dismutase; POD, peroxidase; CAT, catalase; TN, total nitrogen; TP, total phosphorus; TK, total potassium; OM, organic matter; AN, available nitrogen; AP, available phosphorus; AK, available potassium; S-Acp, soil acid phosphatase; S-Cat, soil catalase; S-Sc, soil sucrase; S-Ue, soil urease.

Under controlled greenhouse conditions, inoculation with *Pseudomonas marginalis* A39 was associated with improved drought performance of *Pinus sylvestris* var. *mongolica* seedlings, as summarized in Figure 8. Strain A39 tolerated PEG-induced osmotic stress and retained several plant growth-promoting traits, including ACC deaminase activity, IAA production, siderophore synthesis, and phosphorus solubilization. In the pot experiment, A39-inoculated seedlings showed greater growth, stronger antioxidant enzyme responses, lower ROS and MDA accumulation, improved soil nutrient availability, and enhanced soil enzyme activities compared with non-inoculated controls under drought stress. Rhizosphere bacterial community shifts and genome-inferred functional traits further indicated potential links among A39 inoculation, plant physiological responses, soil biochemical improvement, and microbial community restructuring under water-limited conditions. However, these results should be interpreted as coordinated responses associated with A39 inoculation rather than as direct evidence of root colonization or a confirmed causal mechanism. Future studies involving colonization assays, strain-persistence evaluation, targeted gene functional validation, long-term field trials, and biosafety assessment are needed to verify the ecological stability and application potential of A39 as a candidate PGPR resource for improving drought resilience in forest seedlings.

## Figures and Tables

**Table 1:** Effects of osmotic stress on drought tolerance and plant growth-promoting traits ability of strain A39.

Traits	No Stress (Control)	Desiccation Stress (−0.73 MPa)
Drought tolerance (Absorbance at 600 nm)	1.82 ± 0.02 a	0.83 ± 0.01 b
Siderophore production (SU%)	177.60 ± 5.68 a	102.50 ± 3.33 b
IAA production (μg mL^−1^)	4.16 ± 0.15 a	4.00 ± 0.08 a
ACC deaminase activity (umol mg^−1^ protein h^−1^)	3.22 ± 0.07 a	2.25 ± 0.02 b
Solubilized P (μg mL^−1^)	12.92 ± 3.56 a	5.40 ± 0.57 b

Different lowercase letters indicate significant differences between treatments at *p* < 0.05.

**Table 2:** Pearson correlation analysis between plant physiological parameters and soil biochemical properties.

Parameters		Growth Characteristics	Photosynthetic Characteristics	Soil Characteristics	Soil Enzyme		
					S-Acp	S-Ue	S-Sc
Plant parameters	O_2_^−^	-	−0.88 **	−0.75 *	−0.88 **	−0.82 *	-
	H_2_O_2_	-	−0.85 **	−0.73 *	−0.86 **	-	-
	MDA	-	−0.85 **	−0.74 *	−0.88 **	-	-
	PRO	-	−0.87 **	−0.74 *	−0.91 **	−0.88 **	-
	POD	-	-	-	-	-	-
	SOD	-	-	-	-	-	-
	CAT	-	-	-	-	-	-
Soil parameters	OM	0.79 *	0.85 **	0.91 **	-	0.93 **	0.91 **
	TP	-	0.90 **	0.85 **	-	0.94 **	-
	TK	-	0.85 *	0.91 **	-	0.93 **	-
	AN	0.83 *	0.85 *	0.91 **	-	0.94 **	0.90 **
	AP	-	0.90 **	0.86 **	0.91 **	0.94 **	-
	AK	0.77 *	0.85 **	0.91 **	0.90 **	0.98 **	-
	S-Acp	-	0.89 **	0.81 *	-	0.92 **	-
	S-Ue	0.79 *	0.86 **	0.90 **	-	-	0.89 **
	S-Sc	0.79 *	0.85 **	0.81 *	-	0.89 **	-

* Significantly correlated at the 0.05 level; ** Significantly correlated at the 0.01 level. Growth characteristics include plant height, ground diameter, and weight; photosynthetic characteristics refer to total chlorophyll and carotenoid contents; soil characteristics encompass soil organic matter and nutrient contents (TN, TP, TK, AN, AP, AK); soil enzymes include S-Acp, S-Ue, and S-Sc.

**Table 3:** Representative genes in strain A39 associated with plant growth-promoting traits and drought tolerance.

Functional Category	Representative Gene(s)	Putative Role
Nitrogen metabolism	*nifU, nasA, nirB*	Nitrogen assimilation/fixation-related potential
Phosphate acquisition/carbon metabolism	*pyk*	Carbon metabolism associated with phosphorus-solubilization-related function
IAA synthesis	*iaaM*	Auxin-related plant growth promotion
ACC deaminase/iron acquisition	*acdS; afuA, afuB, afuC*	Stress alleviation and iron transport
Oxidative stress defense	*SOD2, catB*	ROS scavenging
Osmotic adaptation	*kdpA, kdpD; proV, proW, proX; betA, betB; putA*	Ion homeostasis and osmoprotection

## Data Availability

The original data presented in the study are openly available in NCBI GenBank. The 16S rRNA gene sequence of strain A39 is available under accession number MT280205.1. The draft genome assembly of Pseudomonas marginalis A39 has been submitted to NCBI GenBank under accession number JBYVMD000000000, with BioProject PRJNA1474451 and BioSample SAMN60596965.

## Supplementary Materials

The following supporting information can be downloaded at https://www.mdpi.com/article/10.3390/microorganisms14071544/s1, Figure S1: Colony morphology and Gram staining of *Pseudomonas marginalis* A39; Figure S2: Effects of A39 inoculation on (a) carotenoid content, (b) malondialdehyde (MDA) content, and (c) proline (PRO) content in *Pinus sylvestris* var. *mongolica* seedlings under different drought gradients; Figure S3: Rarefaction curves of rhizosphere bacterial communities; Figure S4: Shared and unique OTUs among different treatments; Figure S5: Additional analyses of rhizosphere bacterial communities under different drought gradients; Figure S6: Circular genome map of strain A39; Figure S7: COG functional classification of annotated genes in strain A39; Figure S8: KEGG pathway classification of annotated genes in strain A39; Figure S9: GO functional classification of annotated genes in strain A39; Table S1: Genome-based taxonomic analysis of strain A39; Table S2: PCR Primers for genes related to plant growth-promoting traits and drought stress responses; Table S3: Genome assembly and annotation statistics of *Pseudomonas marginalis* A39; Table S4: Complete list of putative genes in the *Pseudomonas marginalis* A39 genome associated with plant growth-promoting traits and drought tolerance; Table S5: Coefficients of variation (CV, %) for the main measured variables.

## Abbreviations

The following abbreviations are used in this manuscript:

ABA	Abscisic acid
ACC	1-aminocyclopropane-1-carboxylate
ADW	Aboveground dry weight
AK	Available potassium
AN	Available nitrogen
ANI	Average nucleotide identity
ANIb	Average nucleotide identity based on BLAST
ANOVA	Analysis of variance
AP	Available phosphorus
BLAST	Basic Local Alignment Search Tool
CAS	Chrome azurol S
CAT	Catalase
cDNA	Complementary DNA
CFU	Colony-forming units
CK	Control treatment
COG	Cluster of Orthologous Groups
dDDH	Digital DNA-DNA hybridization
DNA	Deoxyribonucleic acid
ELISA	Enzyme-linked immunosorbent assay
ET	Ethylene
GO	Gene Ontology
H_2_O_2_	Hydrogen peroxide
IAA	Indole-3-acetic acid
KEGG	Kyoto Encyclopedia of Genes and Genomes
LD	Light drought
LDA	Linear discriminant analysis
LEfSe	Linear discriminant analysis effect size
MD	Moderate drought
MDA	Malondialdehyde
NB	Nutrient broth
NCBI	National Center for Biotechnology Information
ND	Normal watering
O_2_^−^	Superoxide anion
OD_600_	Optical density at 600 nm
OM	Organic matter
PCA	Principal component analysis
PEG	Polyethylene glycol
PGP	Plant growth-promoting
PGPR	Plant growth-promoting rhizobacteria
POD	Peroxidase
PRO	Proline
qRT-PCR	Quantitative real-time polymerase chain reaction
RNA	Ribonucleic acid
ROS	Reactive oxygen species
S-Acp	Soil acid phosphatase
S-Cat	Soil catalase
S-Sc	Soil sucrase
S-Ue	Soil urease
SD	Severe drought
SMRT	Single-molecule real-time
SOD	Superoxide dismutase
SPSS	Statistical Package for the Social Sciences
SRWC	Soil relative water content
TK	Total potassium
TN	Total nitrogen
TP	Total phosphorus
TYGS	Type (Strain) Genome Server
UDW	Underground dry weight
