# Supplementary material for: Microbial Diversity and Genome Analyses Provide Insights into the Role of Pseudomonas marginalis A39 in Improving Drought Tolerance in Pinus sylvestris var. mongolica
Source: Microorganisms. 2026 Jul 15;14(7):1544. doi: 10.3390/microorganisms14071544 (PMC13414449; doi:10.3390/microorganisms14071544)
Supplement: Supplementary file 1 [file microorganisms-14-01544-s001.zip › microorganisms-4411853-supplementary.pdf]

# *Supplementary Material*

## 1. Supplementary Figures and Tables

1.1. Supplementary Tables

**Supplementary Table S1.** Genome-based taxonomic analysis of strain A39

| Analysis | Reference strain | Value | Aligned genome fraction | Species-level interpretation |
|---|---|---|---|---|
| ANIb | *Pseudomonas marginalis* ICMP 3553[T] | 97.54% | 86.67% | Above species-level threshold |
| ANIb | *Pseudomonas petroselini* MAFF 311094[T] | 97.53% | 84.62% | Above species-level threshold |
| dDDH | *Pseudomonas marginalis* ICMP 3553[T] | 86.5% | — | Above species-level threshold |
| dDDH | *Pseudomonas petroselini* MAFF 311094[T] | 86.0% | — | Above species-level threshold |
| TYGS assignment | — | — | — | Assigned as *Pseudomonas marginalis* |

Note: ANIb, average nucleotide identity based on BLAST; dDDH, digital DNA-DNA hybridization. Genome-based analyses classified strain A39 as *Pseudomonas marginalis*. The 16S rRNA gene accession MT280205.1 was retained only as the initial molecular characterization record.

**Supplementary Table S2.** PCR Primers for genes related to plant growth-promoting traits and drought stress responses

| Gene | Primers | Sequence (5′to3′) |
|---|---|---|
| *pyk* | pyk-F | CGCAGCCGTTCATTTAGCA |
| | pyk-R | TTGTTCTCGTTCCCGTTCC |
| *afuC* | afuC-F | GTTGAGTAACCTCGACGCCA |
| | afuC-R | AGGTTGGCATCGCCCATAAA |
| *catB* | catB-F | GACAAGGAGAACAATTTCCAACAG |
| | catB-R | AGTAGGAGATCCAGATGCCAC |
| *acdS* | ACC1140 | GCTGGTGCAGGAAAACTGGG |
| | ACC1623 | GAARCGCGTRTCGAGCACCAC |
| *SOD2* | SOD2-F | TCGTCCTCCTCATCCTCCTC |
| | SOD2-R | AGACACTTCCAGTGACAGTG |
| *kdpA* | kdpA-in-F | GAGGGCCTAAACGATGTCGTGGAGCAAA |
| | kdpA-in-R | GCGTGCCACCGACATCCCCAGACCGA |
| *putA* | pu | CTCTAGAGTCAGCCGATCGCCATCAG |
| | pd | CGAGCTCGATGAGCCAGACCAGCTTC |
| *iaaM* | iaaM-F | CTTACGAGAAAGGCACGAC |
| | iaaM-R | TAGATGCTGGGCAAACG |

| | | |
|---|---|---|
| *nifU* | nifU-F | CGCGGATCCATGGATTTCTTTGCC |
| | nifU-R | CGGCTCGAGTTATGTATCTTTGTTT |
| *gyrB* | gyrB-F | GTCGTGCGTATCGTGAGCGT |
| | gyrB-R | TGCTGCCGTTGTACACGATT |

[a]The gyrB gene was used as the internal reference gene for qPCR normalization
[b]All primer sequences are presented in the 5' to 3' direction, and the primers were designed to specifically amplify target gene fragments for subsequent expression analysis

**Supplementary Table S3.** Genome assembly and annotation statistics of *Pseudomonas marginalis* A39

| Category | A39 |
|---|---|
| Assembly type | Draft genome |
| Genome size（bp） | 6834719 |
| Total scaffolds | 149 |
| Scaffold N50 (bp) | 114811 |
| Estimated sequencing coverage (×) | 173.8 |
| Average gene length (bp) | 976 |
| N（%） | 0.11 |
| G+C（%） | 58.46 |
| Gene num | 6190 |
| Protein-coding genes | 6007 |
| t RNA genes | 57 |
| rRNA genes | 3 |
| Protein with signal peptides | 881 |
| CRISPRS | 0 |
| eggNOG annotated proteins | 5278 |
| Genes assigned to GOs | 5354 |
| Genes assigned to KEGGs | 6197 |

[a]Coverage was estimated by dividing the clean data size by the draft genome size. [b]COG/eggNOG, GO, and KEGG values indicate the numbers of predicted genes or proteins with functional annotations in the corresponding databases. The 16S rRNA gene sequence of strain A39 has been deposited in GenBank under accession number MT280205; genome submission information is provided in the Data Availability Statement.

**Supplementary Table S4.** Complete list of putative genes in the *Pseudomonas marginalis* A39 genome associated with plant growth-promoting traits and drought tolerance

| Gene name | Ec No. | Gene annotation | Description |
|---|---|---|---|
| *nasA* | EC:1.7.99.- | Nitrogen metabolism | nitrate/nitrite transporter |
| *nirB* | EC:1.7.1.15 | Nitrogen metabolism | nitrite reductase (NADH) large subunit |
| *nifU* | - | Nitrogen metabolism | nitrogen-fixation protein |
| *nirS* | EC:1.7.2.1 | Nitrogen metabolism | nitrite reductase (NO-forming) |
| *cynT* | EC:4.2.1.1 | Nitrogen metabolism | carbonic anhydrase |
| *glnA* | EC:6.3.1.2 | Nitrogen metabolism | glutamine synthetase |
| *ncd2* | EC:1.13.12.16 | Nitrogen metabolism | nitronate monooxygenase |
| *gltD* | EC:1.4.1.13 | Nitrogen metabolism | glutamate synthase small chain |
| *gltB* | EC:1.4.1.13 | Nitrogen metabolism | glutamate synthase large chain |

| | | | |
|---|---|---|---|
| *ntrB* | EC:2.7.13.3 | Two-component system | nitrogen regulation sensor histidine kinase |
| *arcC* | EC:2.7.2.2 | Nitrogen metabolism | carbamate kinase |
| *gdhA* | 1.4.1.4 | Nitrogen metabolism | glutamate dehydrogenase (NADP+) |
| *GDH2* | 1.4.1.2 | Nitrogen metabolism | glutamate dehydrogenase |
| *narI* | 1.7.5.1 | Nitrogen metabolism | nitrate reductase gamma subunit |
| *narH* | 1.7.5.1 | Nitrogen metabolism | nitrite oxidoreductase |
| *norB* | 1.7.2.5 | Nitrogen metabolism | nitric oxide reductase subunit B |
| *pyk* | EC:2.7.1.40 | Glycolysis/Gluconeogenesis | pyruvate kinase |
| *aceB* | EC:2.3.3.9 | Pyruvate metabolism | malate synthase |
| *ppc* | EC:4.1.1.31 | Carbon metabolism | phosphoenolpyruvate carboxylase |
| *pycA* | EC:6.4.1.1 | Citrate cycle (TCA cycle) | pyruvate carboxylase subunit A |
| *gltA* | EC:2.3.3.1 | Glyoxylate metabolism | citrate synthase |
| *aroK* | EC:2.7.1.71 | Phenylalanine, tyrosine and tryptophan biosynthesis | shikimate kinase |
| *pstS* | - | ABC transporters | phosphate transport system substrate-binding protein |
| *pstA* | - | ABC transporters | phosphate transport system permease protein |
| *pstB* | EC:7.3.2.1 | ABC transporters | phosphate transport system ATP-binding protein |
| *GAPDH* | EC1.2.1.12 | Glycolysis/Gluconeogenesis | glyceraldehyde 3-phosphate dehydrogenase |
| *aceE* | EC1.2.4.1 | Glycolysis/Gluconeogenesis | pyruvate dehydrogenase E1 component |
| *gapN* | EC1.2.1.9 | Glycolysis/Gluconeogenesis | glyceraldehyde-3-phosphate dehydrogenase (NADP+) |
| *pgk* | EC2.7.2.3 | Glycolysis/Gluconeogenesis | phosphoglycerate kinase |
| *pgm* | EC5.4.2.2 | Glycolysis/Gluconeogenesis | phosphoglucomutase |
| *yiaY* | EC1.1.1.1 | Glycolysis/Gluconeogenesis | alcohol dehydrogenase |
| *gloA* | EC4.4.1.5 | Pyruvate metabolism | lactoylglutathione lyase |
| *bcpA* | EC4.1.1.112 | Pyruvate metabolism | oxaloacetate decarboxylase |
| *kdpA* | - | Environmental Information Processing | potassium-transporting ATPase subunit |
| *kdpD* | EC:2.7.13.3 | Environmental Information Processing | sensor histidine kinase |
| *kdpC* | - | Environmental Information Processing | potassium-transporting ATPase KdpC subunit |
| *kdpB* | EC:7.2.2.6 | Environmental Information Processing | potassium-transporting ATPase ATP-binding subunit |
| *iaaM* | EC:1.13.12.3 | NAD(P)/FAD-dependent oxidoreductase | IAA-Monooxygenase |
| *atoB* | EC:2.3.1.9 | Tryptophan metabolism | acetyl-CoA C-acetyltransferase |
| *pdhD* | EC:1.8.1.4 | Tryptophan metabolism | dihydrolipoamide dehydrogenase |
| *echA* | EC:4.2.1.17 | Tryptophan metabolism | enoyl-CoA hydratase |
| *amiE* | EC:3.5.1.4 | Tryptophan metabolism | amidase |
| *gcdH* | EC:1.3.8.6 | Tryptophan metabolism | glutaryl-CoA dehydrogenase |
| *hemH* | EC:4.99.1.1 | Porphyrin and chlorophyll metabolism | protoporphyrin/coproporphyrin ferrochelatase |

| | | | |
|---|---|---|---|
| *afuA* | - | ABC transporters | iron(III) transport system substrate-binding protein |
| *afuB* | - | ABC transporters | iron(III) transport system permease protein |
| *afuC* | EC:7.2.2.7 | ABC transporters | iron transport ATP-binding protein |
| *bfr* | EC:1.16.3.1 | Porphyrin and chlorophyll metabolism | bacterioferritin |
| *hemE* | EC:4.1.1.37 | Porphyrin and chlorophyll metabolism | heme o synthase |
| *hemB* | EC:4.2.1.24 | Porphyrin and chlorophyll metabolism | porphobilinogen synthase |
| *treS* | EC:5.4.99.1 | Starch and sucrose metabolism | alpha-amylase |
| *bglX* | EC:3.2.1.21 | Starch and sucrose metabolism | beta-glucosidase |
| *treZ* | EC:3.2.1.141 | Biosynthesis of secondary metabolites | maltooligosyltrehalose trehalohydrolase |
| *lip* | EC:3.1.1.3 | Glycerolipid metabolism | triacylglycerol lipase |
| *SOD2* | EC:1.15.1.1 | Peroxisome | superoxide dismutase |
| *catB* | EC:1.11.1.6 | Tryptophan metabolism | catalase |
| *katG* | EC:1.11.1.21 | Phenylalanine metabolism | |
| *gabD* | EC:1.2.1.16 | Alanine, aspartate and glutamate metabolism | glutarate-semialdehyde dehydrogenase |
| *davT* | EC:2.6.1.48 | Lysine degradation | 5-aminovalerate |
| *kdpD* | EC:2.7.13.3 | Two-component system | sensor histidine kinase |
| *putA* | EC:1.5.5.2 | Biosynthesis of antibiotics | proline dehydrogenase |
| *proV* | EC:7.6.2.9 | ABC transporters | proline transport system ATP-binding protein |
| *proW* | - | Membrane transport | glycine betaine |
| *proX* | - | Membrane transport | proline transport system substrate-binding protein |
| *xdhA* | EC:1.17.1.4 | Purine metabolism | xanthine dehydrogenase small subunit |
| *betA* | EC:1.1.99.1 | Glycine, serine and threonine metabolism | cysteine-S-conjugate beta-lyase |
| *betB* | EC:1.2.1.8 | Glycine, serine and threonine metabolism | betaine-aldehyde dehydrogenase |
| *gdh* | EC:1.4.1.2 | Alanine, aspartate and glutamate metabolism | glucose 1-dehydrogenase |
| *atpA* | EC:7.1.2.2 | Energy metabolism | Na+-transporting ATPase subunit alpha |
| *xdhA* | EC:1.17.1.4 | Nucleotide metabolism | xanthine dehydrogenase small subunit |
| *BADH* | EC:1.1.1.31 | Amino acid metabolism | 3-hydroxyisobutyrate dehydrogenase |
| *gor* | EC:1.8.1.7 | Metabolism of other amino acids | glutathione reductase (NADPH) |
| *acdS* | EC:3.5.99.7 | Cysteine and methionine metabolism | 1-aminocyclopropane-1-carboxylate deaminase |

Ec No. refers to Enzyme Commission number, which classifies enzymes based on their catalytic reactions.

**Supplementary Table S5.** Coefficients of variation (CV, %) for the main measured variables

| Variable category | Variable | CK_30 | A39_30 | CK_45 | A39_45 | CK_60 | A39_60 | CK_80 | A39_80 |
|---|---|---|---|---|---|---|---|---|---|
| Plant growth | Plant height | 1.85 | 1.70 | 2.94 | 4.24 | 3.69 | 5.38 | 3.02 | 2.88 |
| | Ground diameter | 4.15 | 4.38 | 4.82 | 4.06 | 2.79 | 6.43 | 6.36 | 6.94 |
| | AFW | 1.64 | 1.91 | 2.50 | 1.30 | 2.50 | 4.69 | 3.64 | 2.86 |
| | ADW | 1.31 | 1.29 | 4.76 | 3.85 | 4.81 | 4.62 | 3.36 | 3.87 |
| | UFW | 1.90 | 2.78 | 1.32 | 2.33 | 1.35 | 1.28 | 2.65 | 2.50 |
| | UDW | 2.78 | 3.92 | 2.50 | 4.65 | 4.00 | 3.92 | 3.33 | 1.64 |
| Soil nutrients | OM | 4.48 | 5.12 | 5.24 | 4.93 | 6.58 | 5.06 | 6.93 | 5.13 |
| | AN | 2.94 | 2.22 | 2.55 | 2.35 | 2.65 | 2.06 | 2.90 | 2.20 |
| | AP | 6.83 | 5.44 | 3.50 | 5.48 | 6.42 | 4.73 | 9.20 | 6.70 |
| | AK | 2.91 | 2.27 | 2.79 | 1.81 | 2.66 | 2.23 | 2.61 | 2.88 |
| | TN | 0.85 | 0.88 | 0.78 | 0.81 | 0.83 | 0.76 | 1.27 | 1.12 |
| | TP | 1.40 | 1.82 | 0.87 | 1.65 | 1.57 | 1.33 | 1.27 | 1.58 |
| | TK | 2.84 | 3.80 | 2.01 | 3.71 | 2.04 | 1.97 | 3.23 | 1.58 |
| Soil enzyme activities | S_ACP | 0.99 | 0.96 | 0.97 | 1.03 | 0.76 | 1.00 | 0.99 | 0.98 |
| | S_UE | 1.00 | 0.99 | 1.00 | 1.00 | 1.01 | 0.89 | 1.00 | 1.00 |
| | S_SC | 1.64 | 2.22 | 2.39 | 3.34 | 2.03 | 2.75 | 1.45 | 1.77 |
| | S_CAT | 1.40 | 1.21 | 1.00 | 1.05 | 1.00 | 0.97 | 1.00 | 1.00 |
| Physiological traits | Total chlorophyll | 3.33 | 4.72 | 4.01 | 5.79 | 5.81 | 5.75 | 5.46 | 5.64 |
| | Carotenoid | 2.04 | 1.40 | 0.67 | 0.96 | 0.72 | 1.00 | 0.93 | 0.88 |
| | Superoxide | 1.10 | 0.99 | 1.00 | 1.13 | 1.00 | 1.10 | 1.00 | 1.01 |
| | $H_2O_2$ | 1.22 | 1.14 | 1.00 | 1.18 | 1.01 | 1.28 | 1.01 | 1.02 |
| | MDA | 2.67 | 4.59 | 4.35 | 4.28 | 4.35 | 5.01 | 5.66 | 6.42 |
| | PRO | 2.57 | 3.21 | 2.85 | 3.90 | 5.27 | 4.17 | 4.29 | 5.27 |
| | SOD | 4.23 | 4.03 | 3.60 | 3.84 | 2.68 | 4.43 | 3.52 | 5.99 |
| | POD | 4.63 | 2.36 | 2.37 | 2.30 | 4.50 | 3.65 | 5.62 | 4.54 |
| | CAT | 3.47 | 3.62 | 4.03 | 3.36 | 4.59 | 5.18 | 4.71 | 2.99 |
| qRT-PCR | *pyk* | 5.71 | 1.26 | 3.24 | 1.19 | 2.28 | 1.05 | 2.55 | 3.86 |
| | *nifU* | 6.00 | 2.20 | 3.92 | 3.05 | 4.86 | 5.00 | 1.96 | 13.71 |
| | *SOD2* | 8.57 | 1.65 | 1.30 | 1.50 | 1.82 | 4.22 | 1.44 | 4.55 |
| | *catB* | 9.47 | 2.83 | 1.74 | 1.72 | 2.01 | 2.51 | 2.21 | 5.43 |
| | *acdS* | 5.31 | 1.95 | 3.09 | 2.00 | 1.96 | 6.04 | 2.80 | 6.29 |
| | *kdpA* | 9.32 | 8.31 | 3.60 | 7.36 | 9.27 | 6.67 | 9.67 | 7.79 |
| | *putA* | 2.27 | 3.27 | 4.24 | 6.00 | 7.32 | 3.82 | 9.52 | 4.59 |
| | *iaaM* | 7.31 | 5.87 | 9.49 | 7.74 | 5.96 | 3.83 | 7.97 | 9.86 |
| | *afuC* | 6.88 | 5.21 | 5.60 | 6.34 | 2.95 | 6.79 | 4.42 | 4.64 |

CV was calculated as standard deviation divided by mean × 100% for each treatment and variable.

1.2. Supplementary Figures

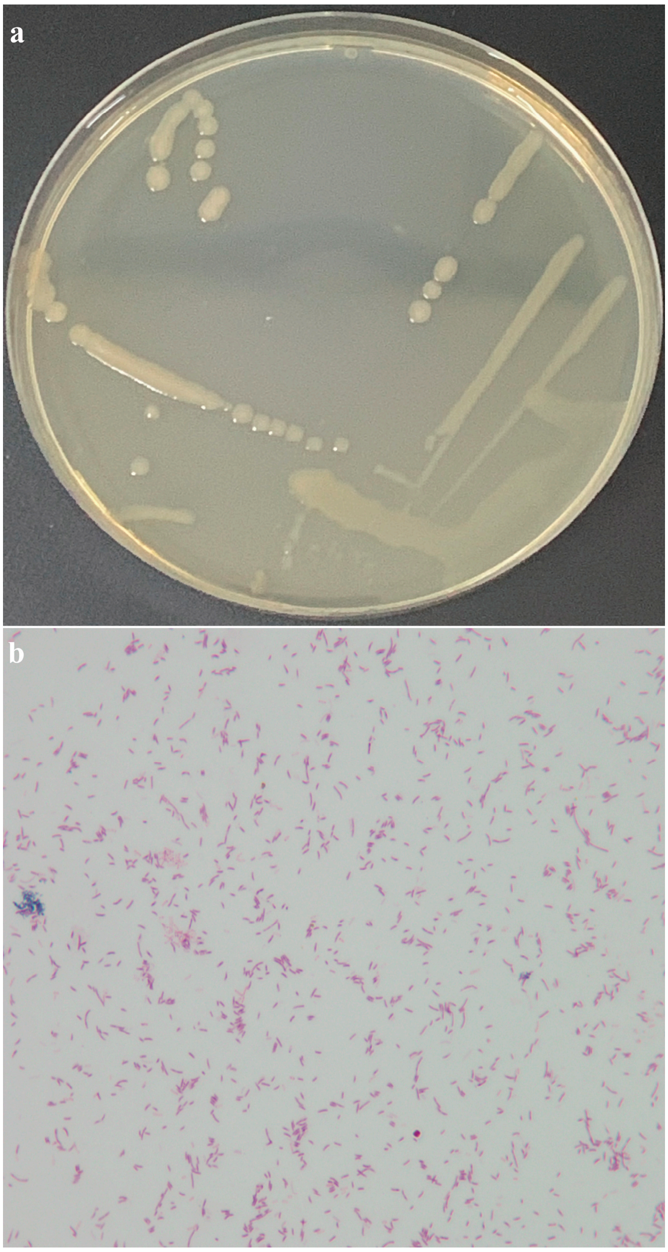


**Supplementary Figure S1.** Colony morphology and Gram staining of *Pseudomonas marginalis* A39. **(a)** Colony morphology of strain A39 on NA medium after incubation at 30 ± 1 °C for 24–48 h, showing creamy-white, round, and smooth colonies. **(b)** Gram-stained cells of strain A39 observed under a light microscope, showing rod-shaped cells with Gram-negative staining characteristics.

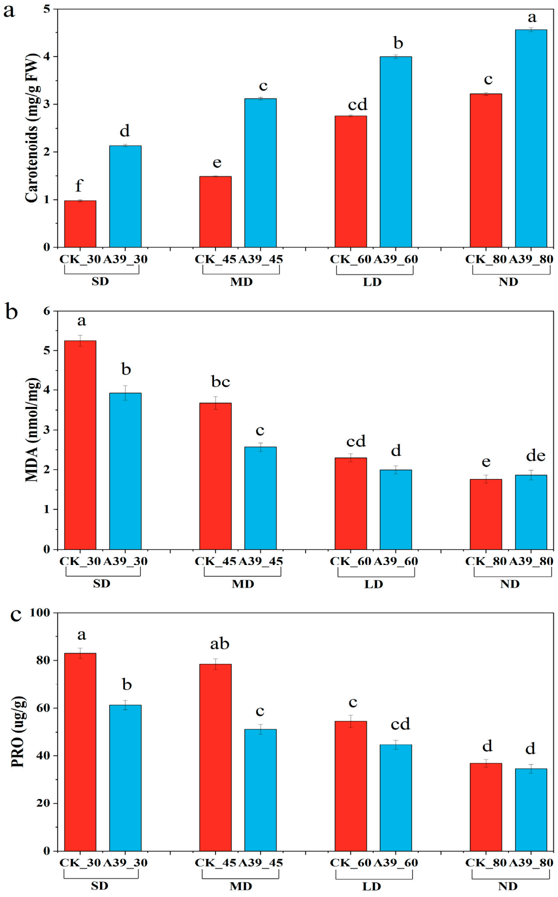


**Supplementary Figure S2.** Effects of A39 inoculation on **(a)** carotenoid content, **(b)** malondialdehyde (MDA) content, and **(c)** proline (PRO) content in *Pinus sylvestris* var. *mongolica* seedlings under different drought gradients. Values are presented as mean ± standard error (n=3). Different lowercase letters indicate significant differences among treatments at *P*<0.05.

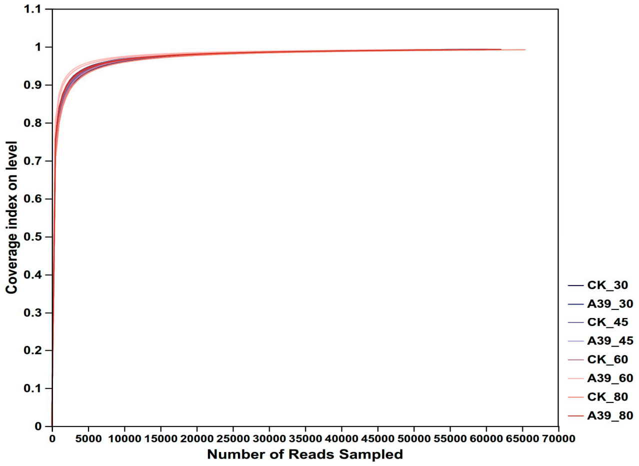


**Supplementary Figure S3.** Rarefaction curves of rhizosphere bacterial communities

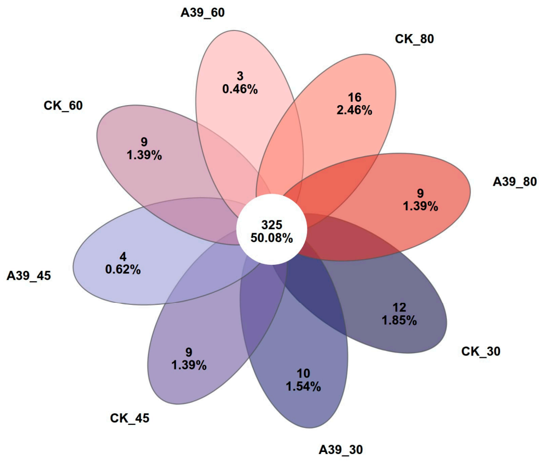


**Supplementary Figure S4.** Shared and unique OTUs among different treatments

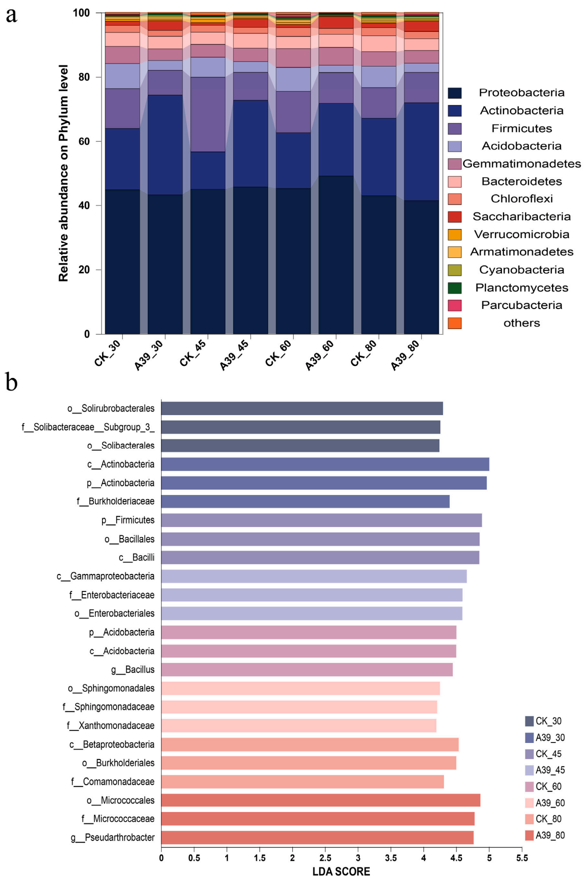


**Supplementary Figure S5.** Additional analyses of rhizosphere bacterial communities under different drought gradients. **(a)** Relative abundance of dominant bacterial phyla. **(b)** LEfSe analysis of differential bacterial taxa. CK and A39 indicate non-inoculated and A39-inoculated treatments, respectively; 30, 45, 60, and 80 correspond to SD, MD, LD, and ND, respectively.

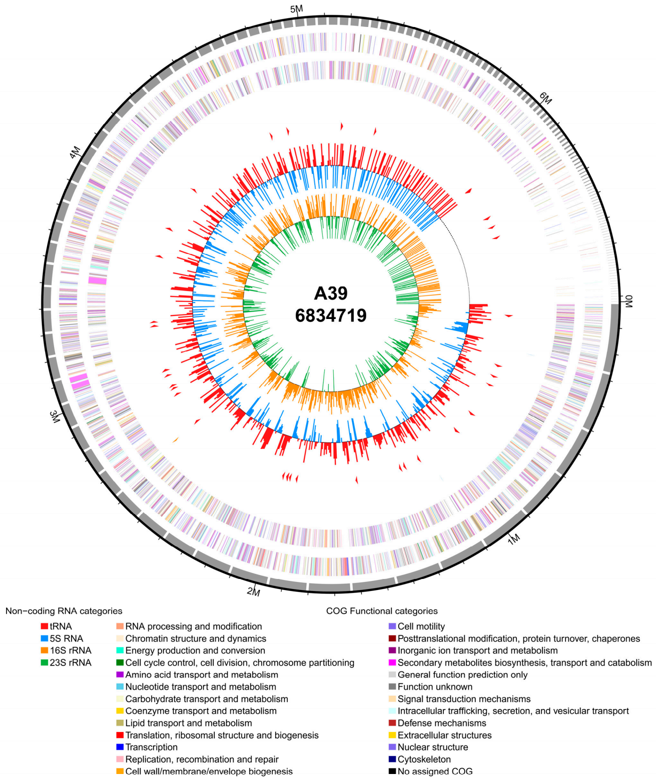


**Supplementary Figure S6.** Circular genome map of strain A39

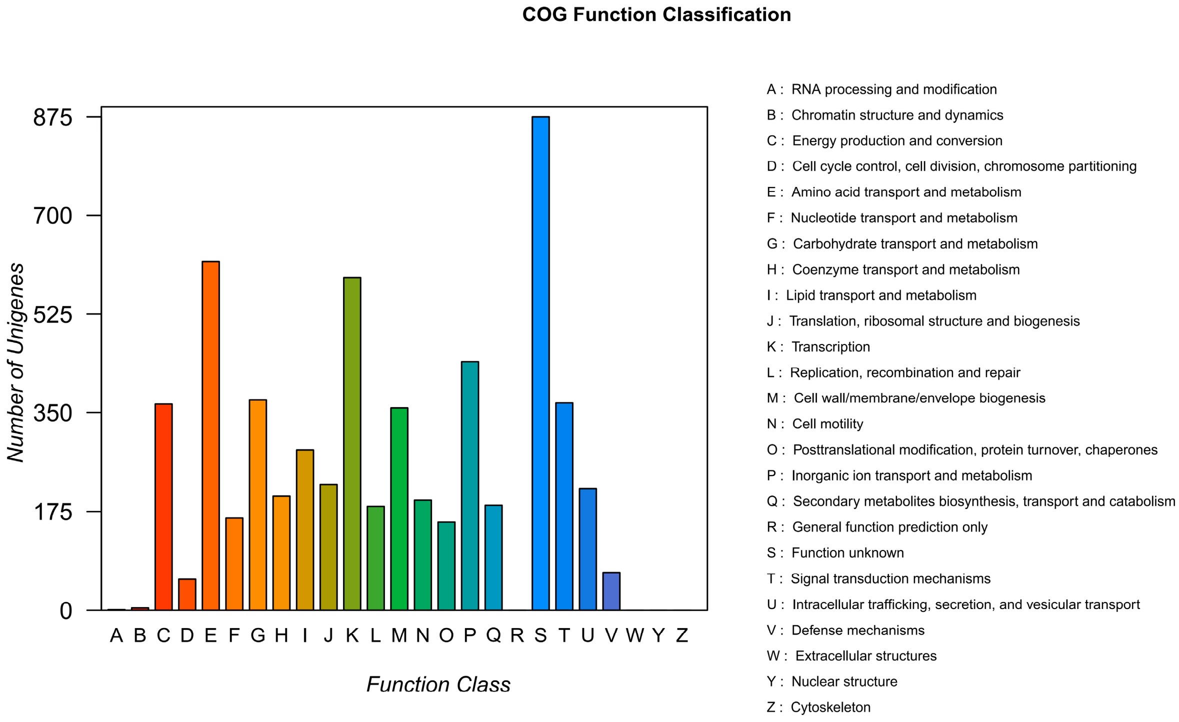


**Supplementary Figure S7.** COG functional classification of annotated genes in strain A39

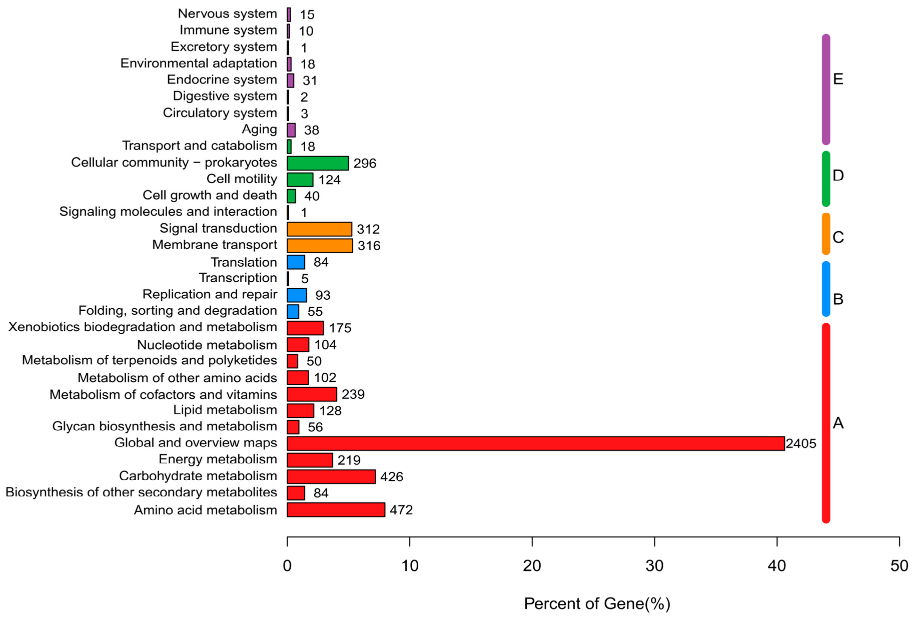


**Supplementary Figure S8.** KEGG pathway classification of annotated genes in strain A39

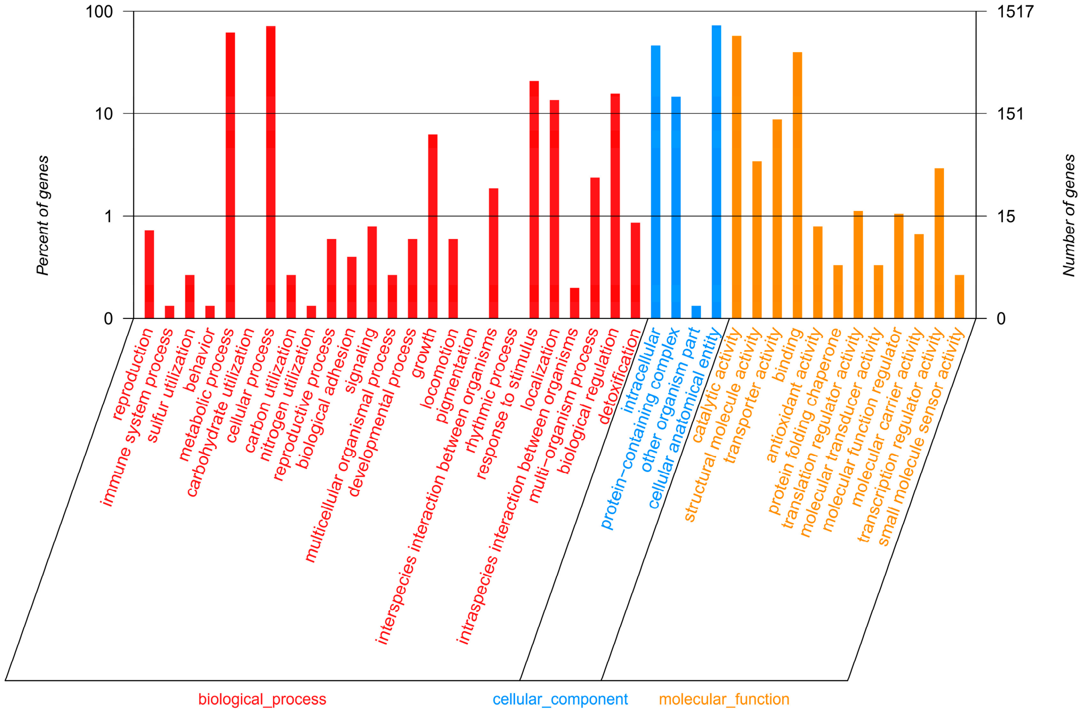


**Supplementary Figure S9.** GO functional classification of annotated genes in strain A39
